# Supplementary material for: The Shc1 adaptor simultaneously balances Stat1 and Stat3 activity to promote breast cancer immune suppression
Source: Nat Commun. 2017 Mar 9;8:14638. doi: 10.1038/ncomms14638 (PMC5347092; doi:10.1038/ncomms14638)
Supplement: Supplementary Information — Supplementary Figures, Supplementary Tables and Supplementary References [file ncomms14638-s1.pdf]

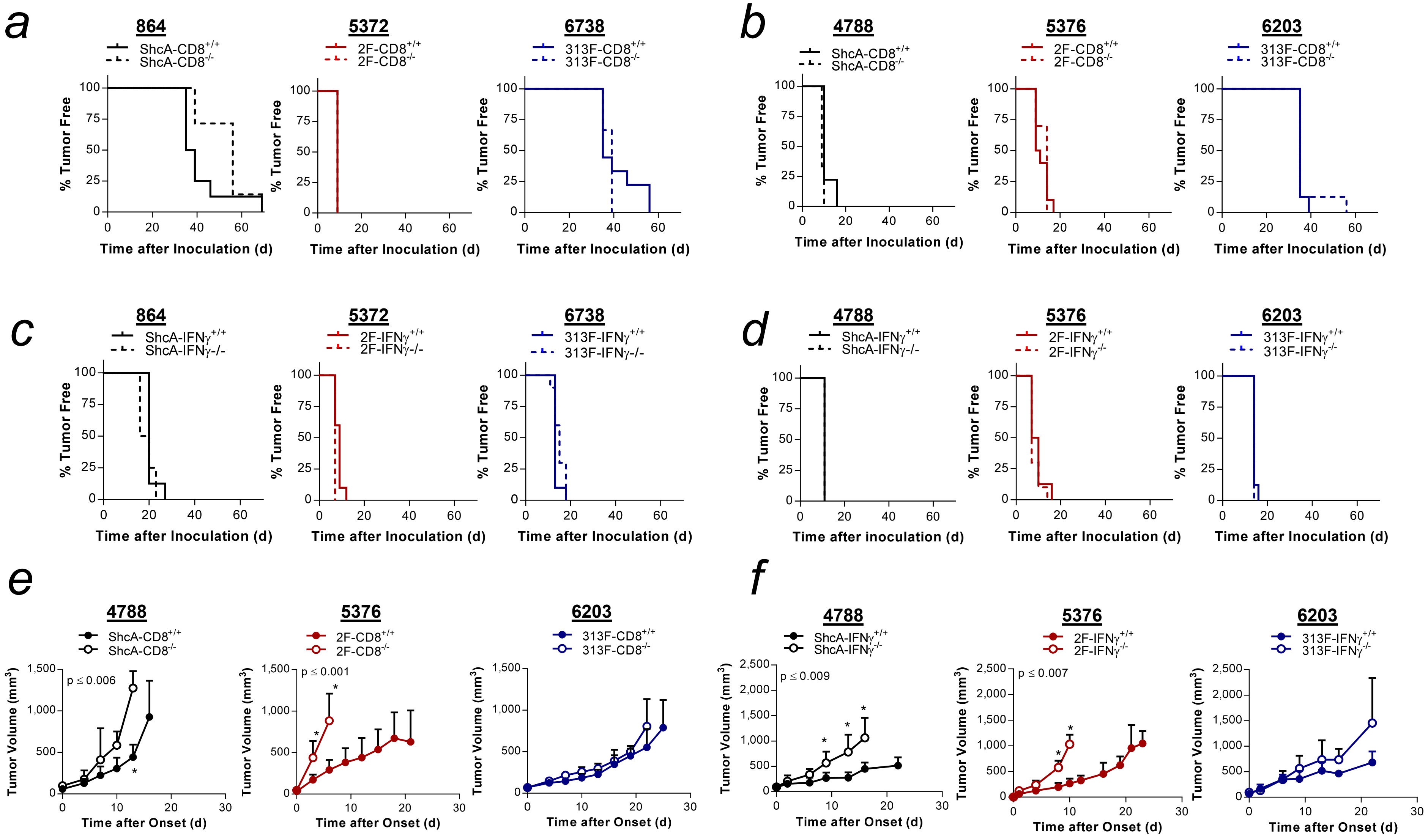

Supplementary Figure 1. Loss of tumor cell autonomous Y239/240-ShcA signaling sensitizes tumors to cytotoxic T cell and IFNγ driven anti-tumor immunity. Kaplan-Meyer curve of first tumor onset (by physical palpation) following injection of the following MT-transformed breast cancer cell lines ShcA (864, 4788), Shc2F (5372, 5376) Shc313F (6203, 6738) into fourth mammary fat pad of (**a, b**) $CD8^{+/+}$ or $CD8^{-/-}$ and (**c, d**) $IFN\gamma^{+/+}$ or $IFN\gamma^{-/-}$ mice. Data are shown as the percentage of tumor free mammary glands following mammary fat pad injection (days) and are representative of n=7-10 tumors per group. Bold lines represent cohort of tumors injected into immune competent $CD8^{+/+}$ or $IFN\gamma^{+/+}$ and dotted lines represent those injected into immunodeficient $CD8^{-/-}$ or $IFN\gamma^{-/-}$ animals. (**e, f**) Tumor outgrowth was monitored by bi-weekly caliper measurements. Tumor growth after first physical palpation is represented as mean tumor volume (mm^3) ± SEM (n=7-10) per group. Significance was determined by multiple t test with Holm-Sidak method for **e** and **f**. *denotes statistically significant time points as indicated in the top left corner.

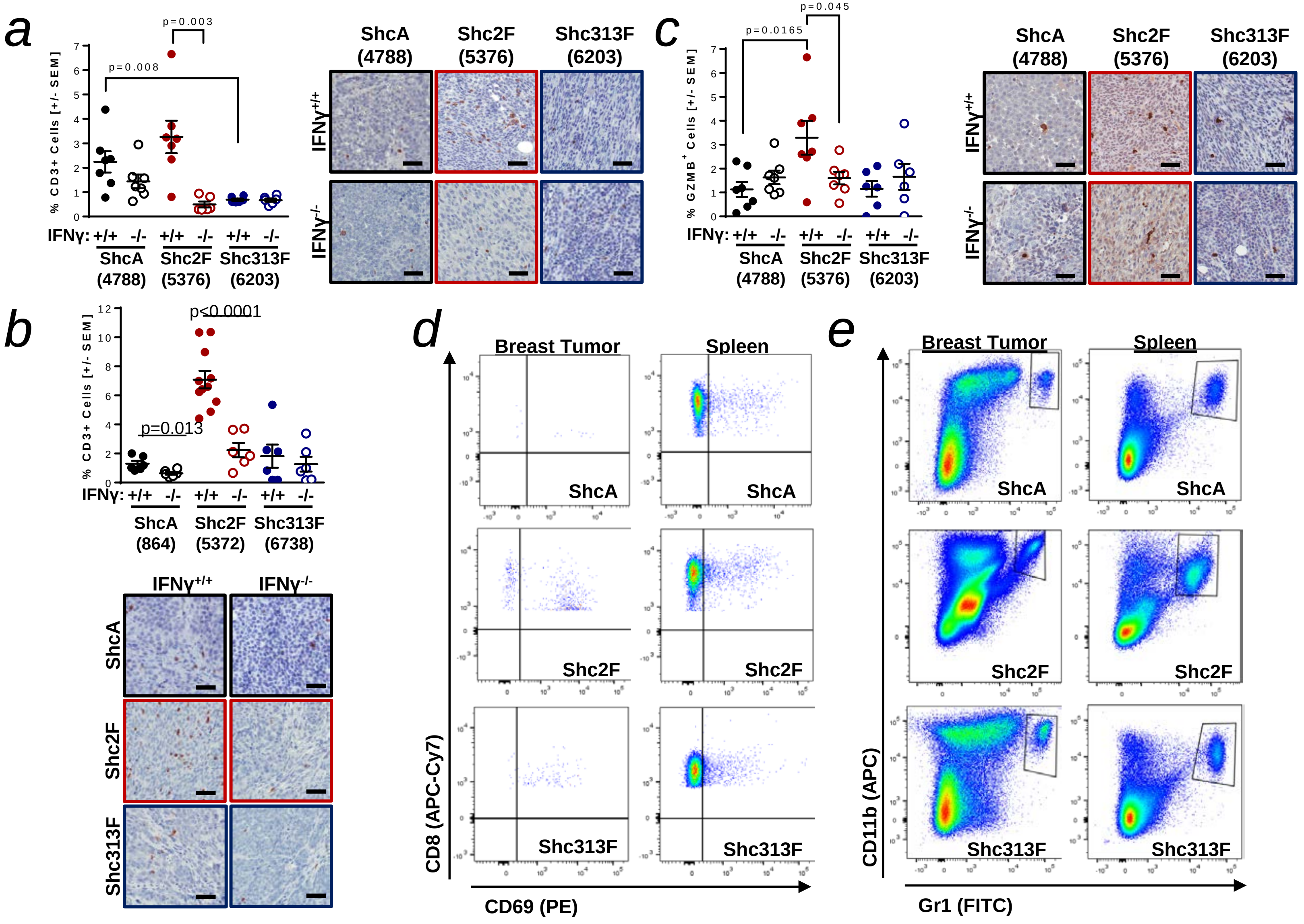

Supplementary Figure 2. Loss of Y239/240-ShcA signaling increases cytotoxic T cell infiltration into mammary tumors. (**a, b**) CD3ε immunohistochemical (IHC) staining of paraffin-embedded sections (n = 6-10/group) isolated from independent ShcA (864, 4788), Shc2F (5372, 5376) and Shc313F (6738, 6203) mammary tumors that emerged in an IFNγ$^{+/+}$ or IFNγ$^{-/-}$ background. The data are represented as percentage of CD3^{+} cells ± SEM. (**c**) Left panel, Tumor sections (n = 6-10) were subjected to Granzyme B (GZMB) IHC staining. The data are represented as percentage of GZMB^{+} cells ± SEM. Right panel shows representative IHC images. Scale bar=50µm (**d, e**) ShcA (864), Shc2F (5372) and Shc313F (6738) breast tumors and matching spleens were harvested from syngeneic (FVB) mice, dissociated and subjected to flow cytometry. Representative dot plots of (**d**) CD8^{+} and CD8^{+}CD69^{+} cytotoxic T cells and (**e**) CD11b^{+}Gr1^{+} cell population (gated) are shown. Significance was determined by Wilcoxon rank-sum test for **a, b** and **c**.

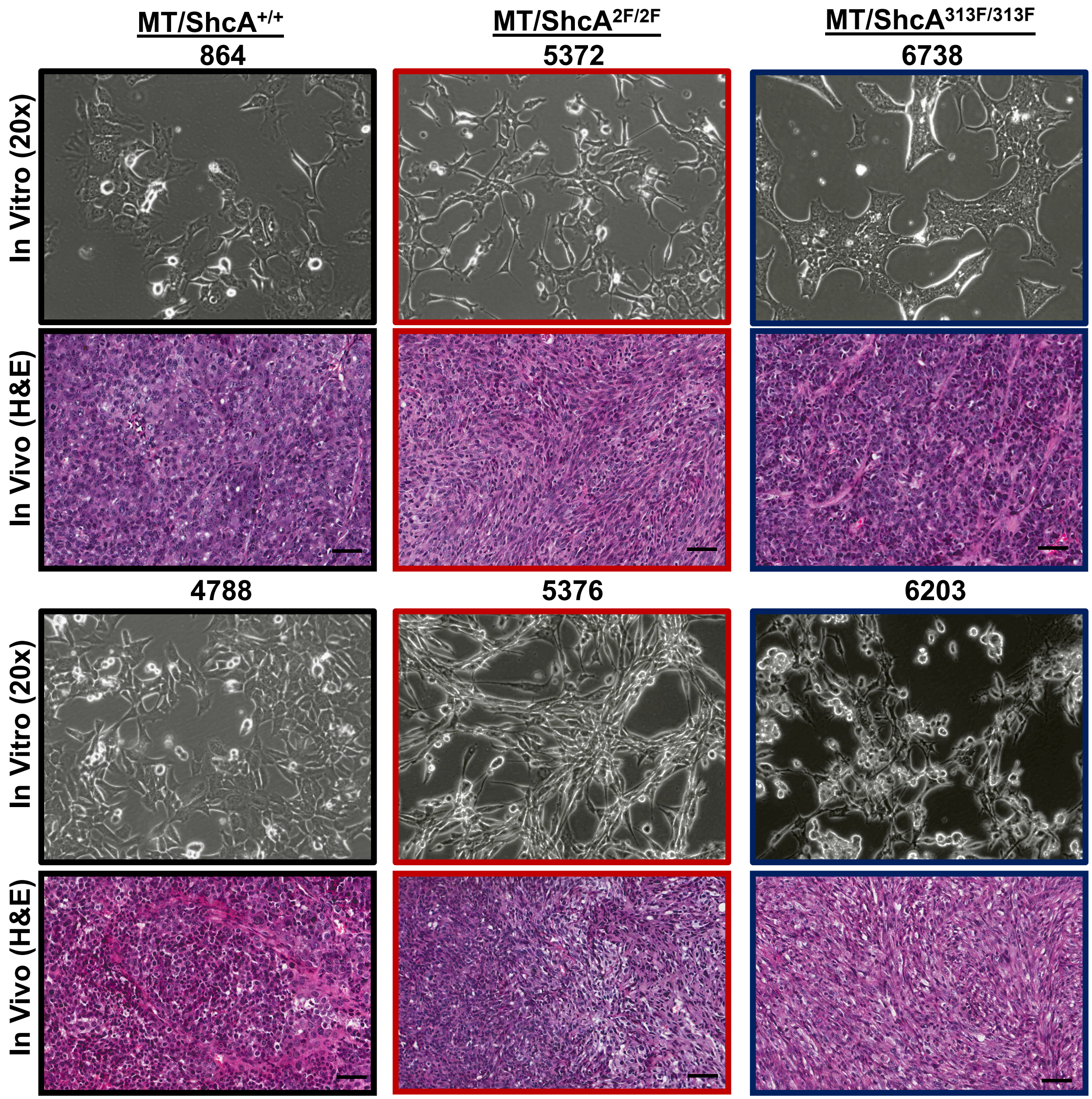

Supplementary Figure 3. The immunosuppressive potential of individual breast cancer cell lines does not correlate with cell morphology or histology of mammary tumors. The morphology of two independent MT/ShcA$^{+/+}$ (864, 4788), MT/Shc$^{2F/2F}$ (5372, 5376) and MT/Shc$^{313F/313F}$ (6203, 6738) was evaluated by phase contrast microscopy (20X magnification). Each cell line was injected into the mammary fat pads of FVB mice and the histology of resulting tumors was evaluated in H&E stained sections. Scale bar=50 microns.

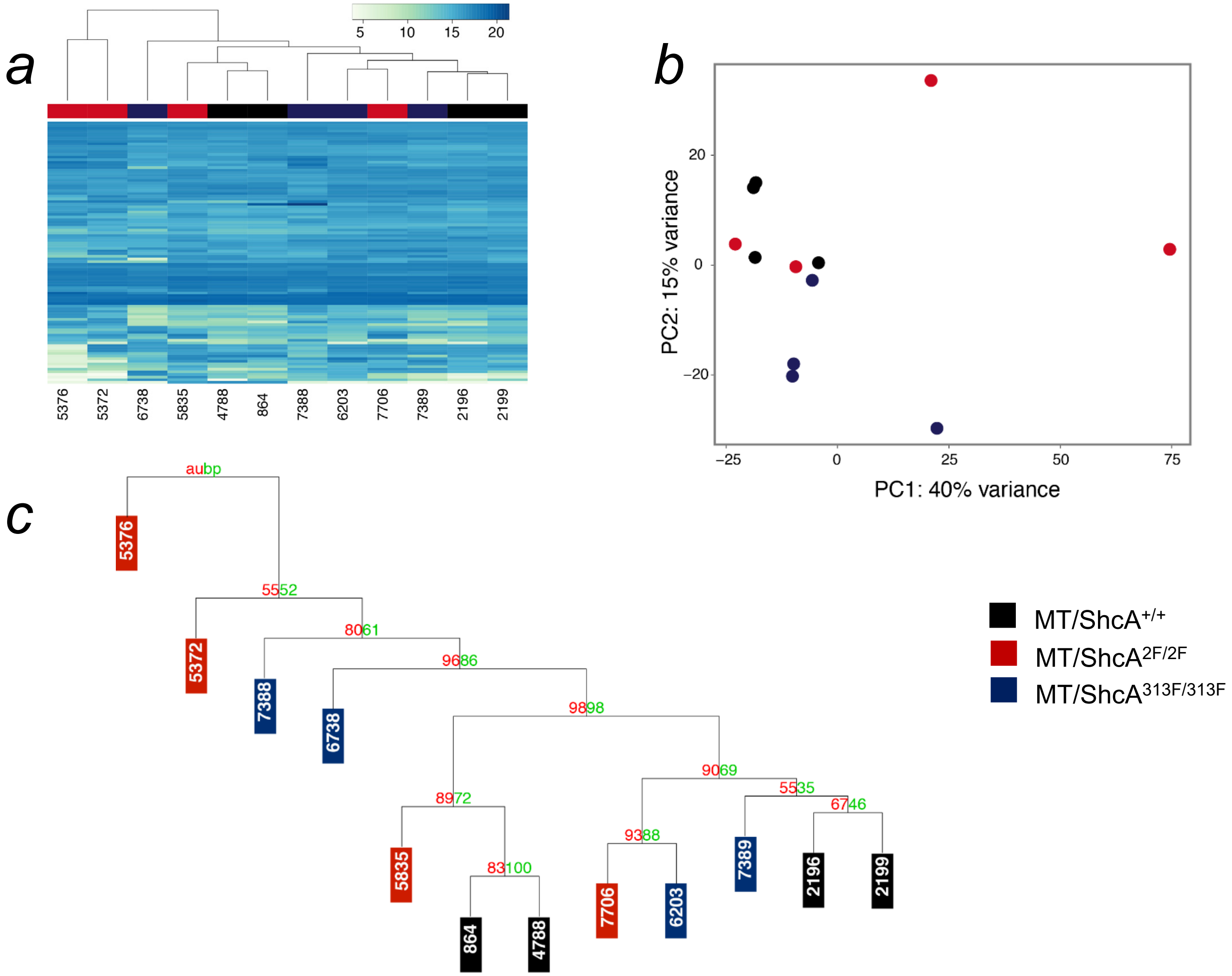

Supplementary Figure 4. Loss of ShcA-driven phospho-tyrosine signaling does not alter the global transcriptome of independent breast cancer cell lines. Global effects of ShcA mutations were evaluated by unsupervised clustering analysis of transcripts expressed in ShcA (864, 4788, 2196, 2199), 2F (5372, 5376, 5835, 7706) and 313F (6203, 6738, 7388, 7389) primary breast cancer cells as assessed by RNA sequencing. We observe that ShcA mutation status does not robustly segregate cell lines based on expression profiles. Normalized, variant stabilized transformed data was used. Shown are the results based on the 1,000 most variant genes. (**a**) Hierarchical clustering of samples. (**b**) Principal component analysis (PCA). (**c**) Multiscale bootstrapping of gene expression clustering. In red, the approximately unbiased (AU) *p*-value is represented. Bootstrapping was performed based on 1,000 iterations.

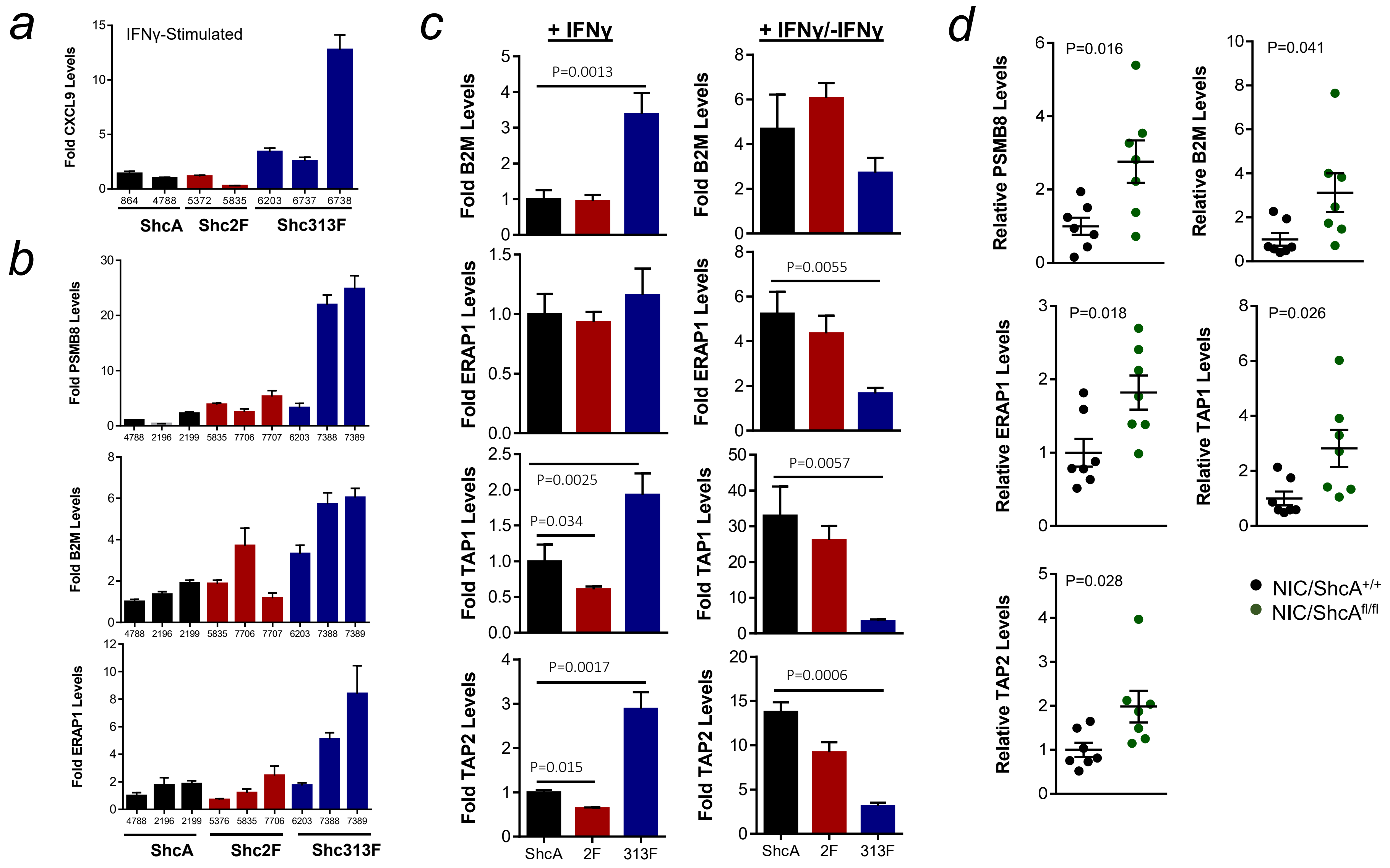

Supplementary Figure 5. pY313-ShcA deficiency augments IFNγ-driven anti-tumor immune responses in mammary tumors. (**a**) MT/ShcA$^{+/+}$, MT/Shc$^{2F/2F}$ and MT/Shc$^{313F/313F}$ cell lines were stimulated with IFNγ (1ng/ml) for 24 hr. Steady state *CXCL9*/*TBP* mRNA levels were assessed by RT-qPCR. The data is shown as the average fold change relative to MT/ShcA$^{+/+}$ (864) ± SD (n=4 replicates each). (**b**) MT/ShcA$^{+/+}$, MT/Shc$^{2F/2F}$ and MT/Shc$^{313F/313F}$ cell lines were assessed for mRNA expression levels of APP machinery components (*PSMB8*, *B2M*, and *ERAP1* relative to *GAPDH*) by RT-qPCR. The data is shown as average fold change relative to MT/ShcA$^{+/+}$ cells (864) ± SD (n=4 replicates each). (**c**) Relative *B2m*, *ERAP1*, *TAP1* and *TAP2* mRNA levels (normalized to *GAPDH*) following 24 hr of IFNγ treatment (1ng/ml) in MT/ShcA$^{+/+}$ (864), MT/Shc$^{2F/2F}$ (5372) and MT/Shc$^{313F/313F}$ (6738) cells. The data is shown as average fold change relative to MT/ShcA$^{+/+}$ cells ± SD (n=12 per condition from three independent experiments). (**d**) RT-qPCR was carried out on RNA isolated from NIC/ShcA$^{+/+}$ and NIC/ShcA$^{fl/fl}$ mammary tumors. Relative fold changes in *PSMB8*, *B2M*, *ERAP1*, *TAP1* and *TAP2* mRNA levels (normalized to *GAPDH*) ± SEM (n=7 tumors per genotype). Significance was determined by two tailed two sample t test for **c** and by Wilcoxon rank-sum test for **d**.

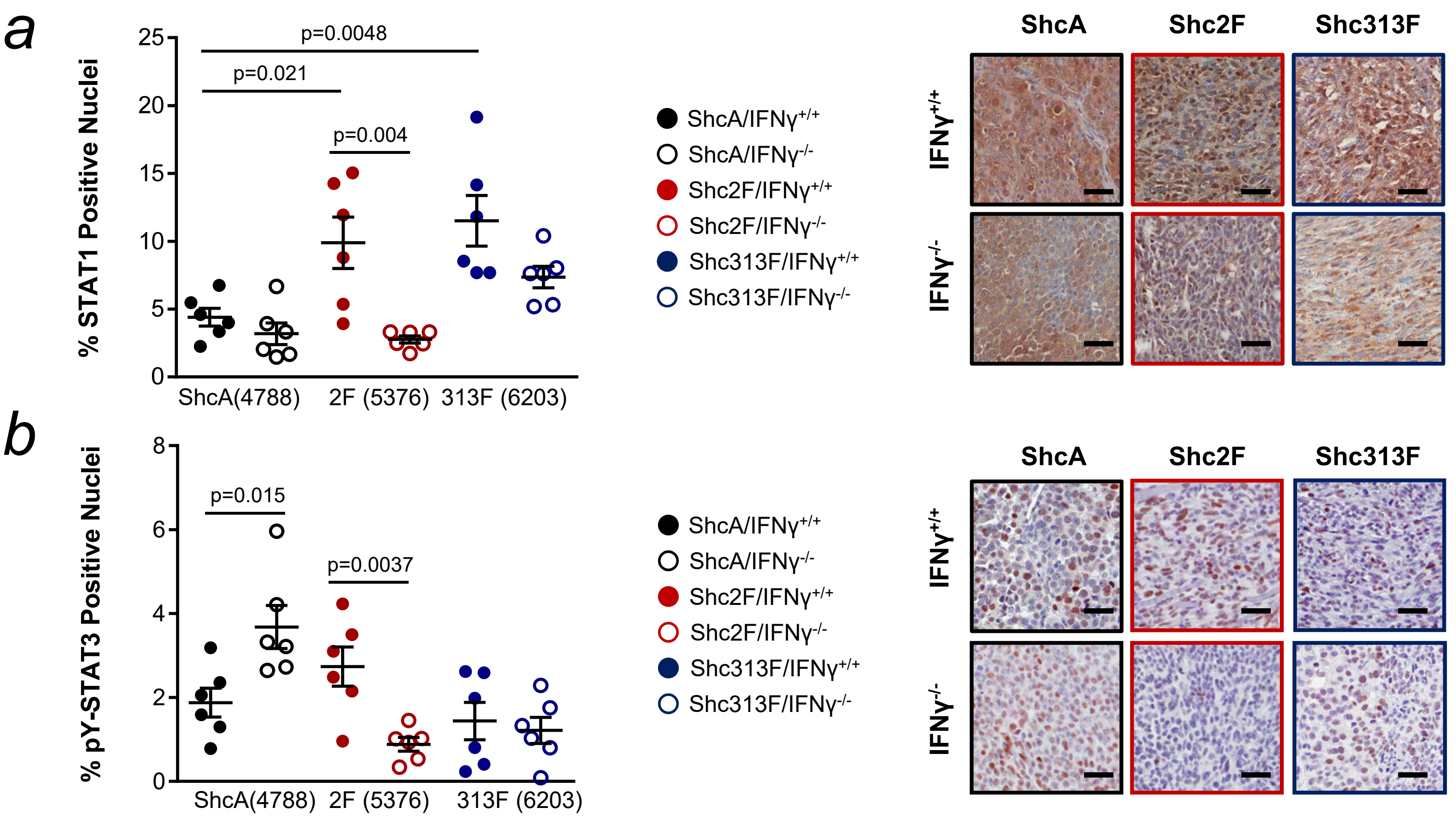

Supplementary Figure 6. Differential activation of the STAT1 and STAT3 pathways in independent breast cancer cell lines and mammary tumors. (**a, b**) STAT1 and pY705-STAT3 immunohistochemical (IHC) analysis of independent MT/ShcA$^{+/+}$ (4788), MT/Shc$^{2F/2F}$ (5376) and MT/Shc$^{313F/313F}$ (6203) mammary tumors harvested from IFNγ$^{+/+}$ or IFNγ$^{-/-}$ mice. Left panels: Percentage of (**a**) STAT1 positive- and (**b**) pY705-STAT3 positive nuclei ± SEM (n=6) Right panels: representative IHC images of stained slides. Significance was determined by Wilcoxon rank-sum test. Scale bar=50μm

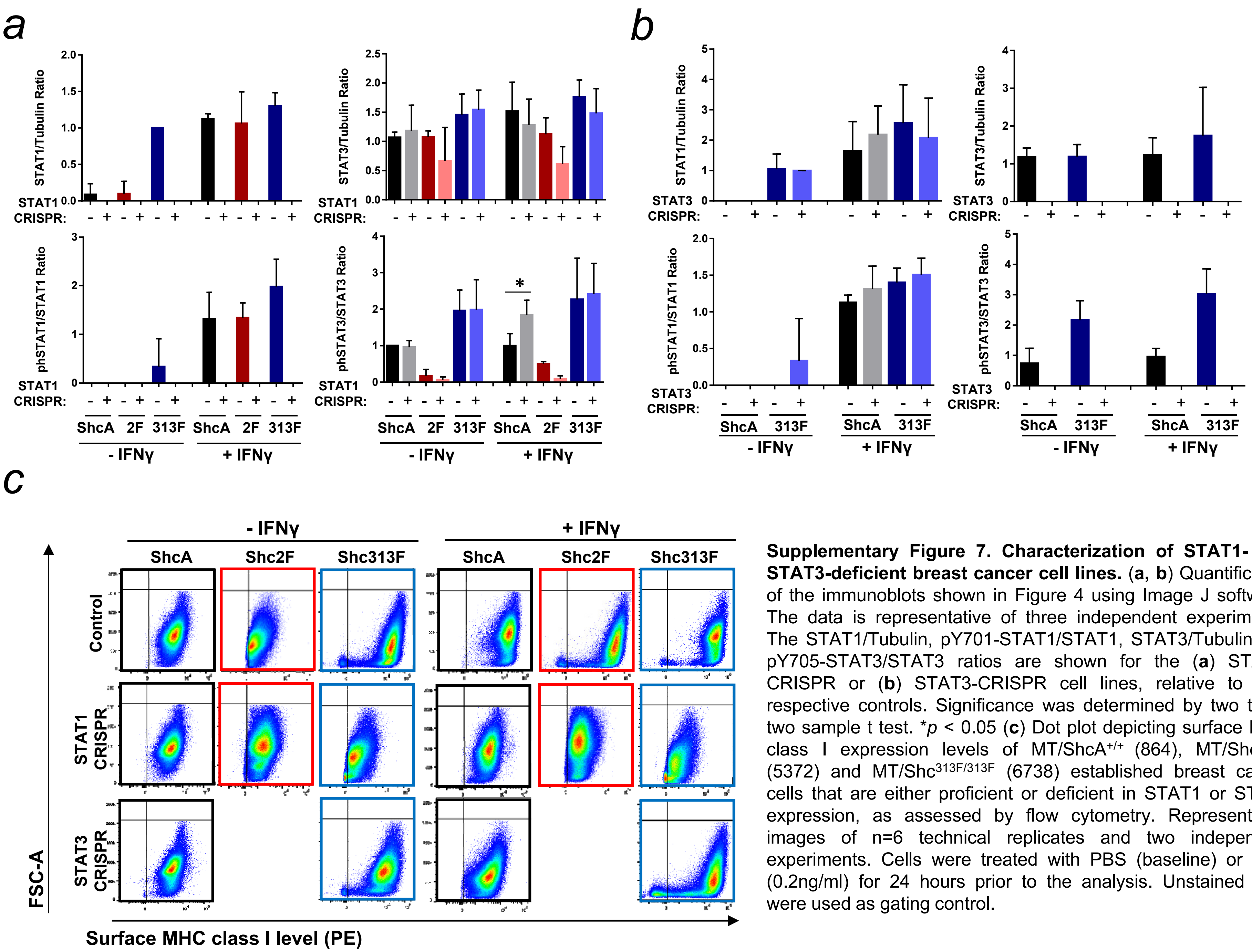

Supplementary Figure 7. Characterization of STAT1- and STAT3-deficient breast cancer cell lines. (**a, b**) Quantification of the immunoblots shown in Figure 4 using Image J software. The data is representative of three independent experiments. The STAT1/Tubulin, pY701-STAT1/STAT1, STAT3/Tubulin and pY705-STAT3/STAT3 ratios are shown for the (**a**) STAT1-CRISPR or (**b**) STAT3-CRISPR cell lines, relative to their respective controls. Significance was determined by two tailed two sample t test. *$p < 0.05$ (**c**) Dot plot depicting surface MHC class I expression levels of MT/ShcA$^{+/+}$ (864), MT/Shc$^{2F/2F}$ (5372) and MT/Shc$^{313F/313F}$ (6738) established breast cancer cells that are either proficient or deficient in STAT1 or STAT3 expression, as assessed by flow cytometry. Representative images of n=6 technical replicates and two independent experiments. Cells were treated with PBS (baseline) or IFNγ (0.2ng/ml) for 24 hours prior to the analysis. Unstained cells were used as gating control.

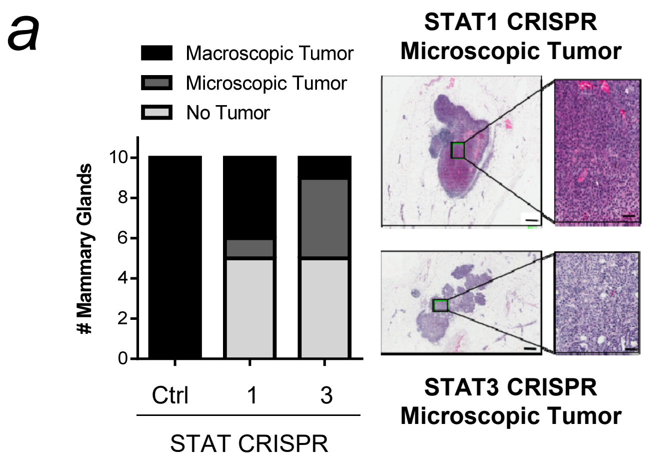

Supplementary Figure 8. *STAT1* and *STAT3* loss in ShcA-proficient breast cancer cells inhibits tumor formation in an immune-regulated manner. (**a**) Control MT/ShcA$^{+/+}$ (864) breast cancer cells, along with *STAT1*- or *STAT3*- deficient pooled clones were injected into the mammary fat pads of syngeneic FVB mice. At the experimental endpoint for the Control-CRISPR group, all animals were sacrificed and the tumor burden (no tumor, unpalpable microscopic lesions or macroscopic tumors) in each mammary gland were determined by H&E staining. Representative images of microscopic lesions are shown. Scale bar=50μm (**b**, **c**) Top panel, mammary tumors derived from MT/ShcA$^{+/+}$ (864), MT/Shc$^{313F/313F}$ (6738) and MT/Shc$^{2F/2F}$ (5372) established cell lines which were stably deleted of *STAT1* or *STAT3* (as indicated), along with corresponding vector controls, were subjected to (**b**) CD3^{+} or (**c**) GZMB^{+} immunohistochemical staining in paraffin-embedded sections. The data is shown as percentage of positively stained cells ± SEM and is representative of 4-8 mammary tumors. Scale bar=50μm. Microscopic *STAT1* and *STAT3* null breast cancer lesions were also evaluated and are highlighted by grey shading. Quantification was performed using Image Scope software. ML = microscopic lesion. BT = breast tumor.

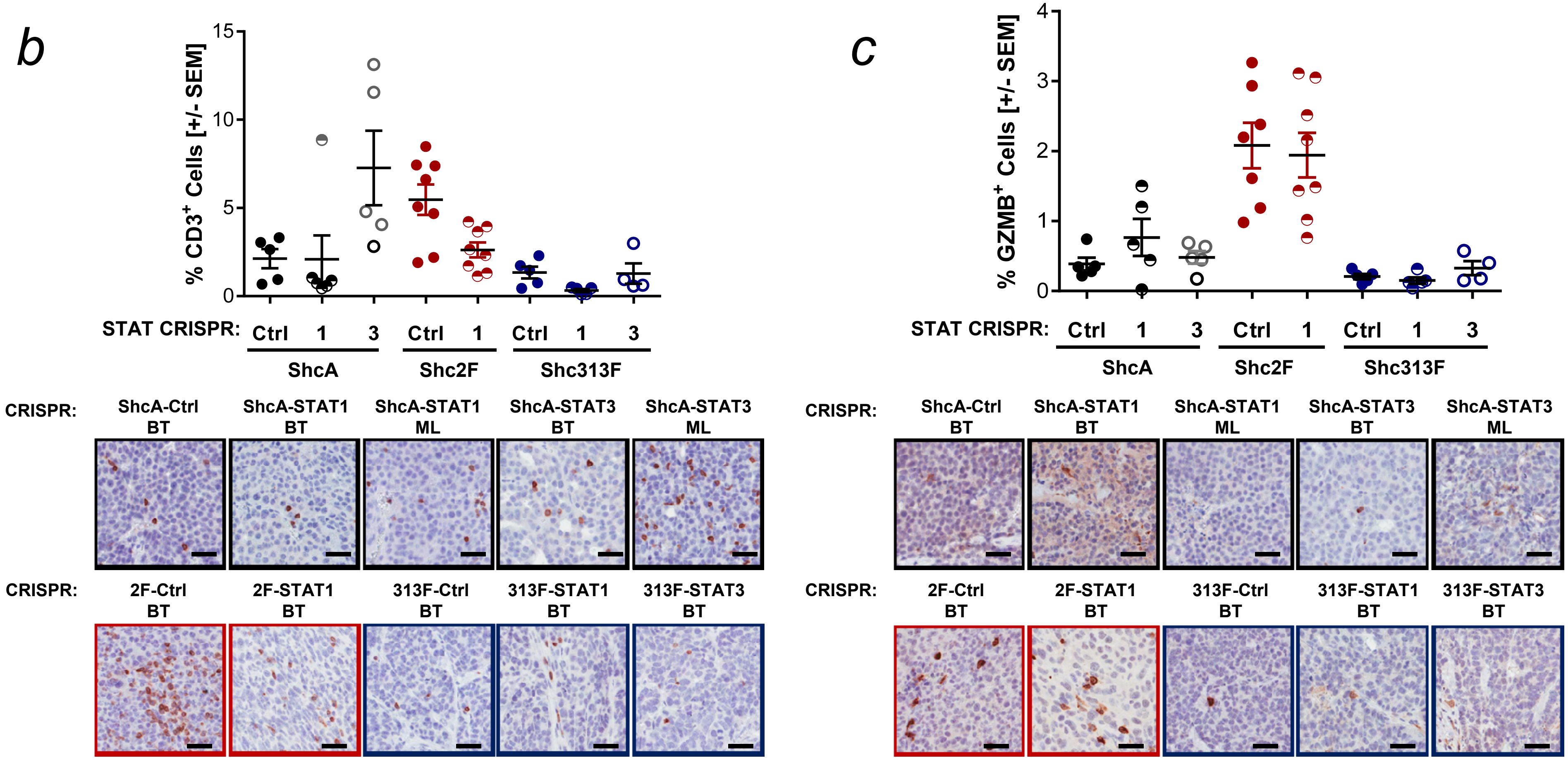

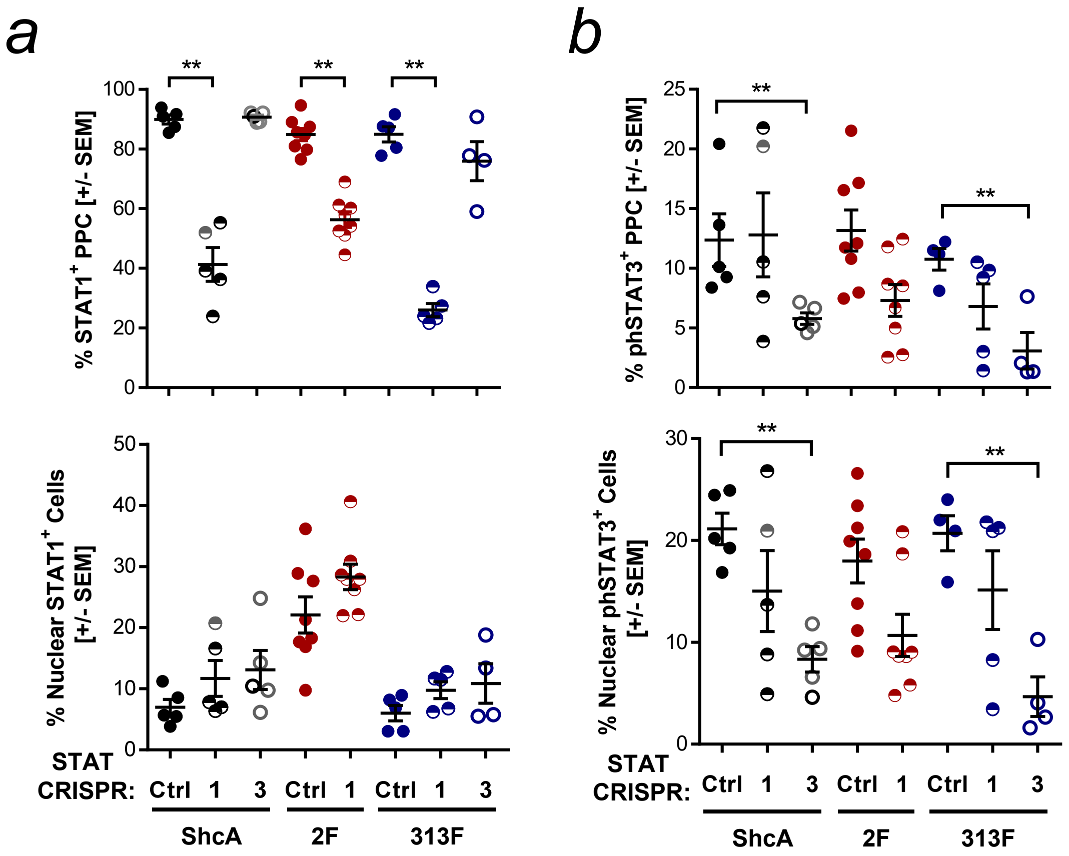

Supplementary Figure 9. Confirmation of mammary epithelial STAT1 and STAT3 loss in breast tumors in vivo. (**a, b**) Control, STAT1- CRISPR or STAT3- CRISPR mammary tumors of the indicated genotypes (MT/ShcA$^{+/+}$ (864), MT/Shc$^{2F/2F}$ (5372), MT/Shc$^{313F/313F}$ (6738)), which emerged in an immunocompetent (FVB) background were analyzed for (**a**) STAT1 and (**b**) pY705-STAT3 levels by immunohistochemical staining of paraffin-embedded sections. For each panel, the top graph represents the average percentage of positive pixels (PPC) per total epithelial area ± SEM and is representative of 4-8 mammary tumors. The bottom graph represents the average % positively-stained nuclei per total epithelial area ± SEM and is representative of 4-8 mammary tumors. Microscopic STAT1 and STAT3 null breast cancer lesions were also evaluated and are highlighted by grey shading. Statistical analysis was performed using Wilcoxon rank-sum test (**p* < 0.01). (**c**) Representative images of immunohistochemical staining of microscopic lesions (ML; non-palpable) and breast tumors (BT) analyzed in **a** and **b**.

C

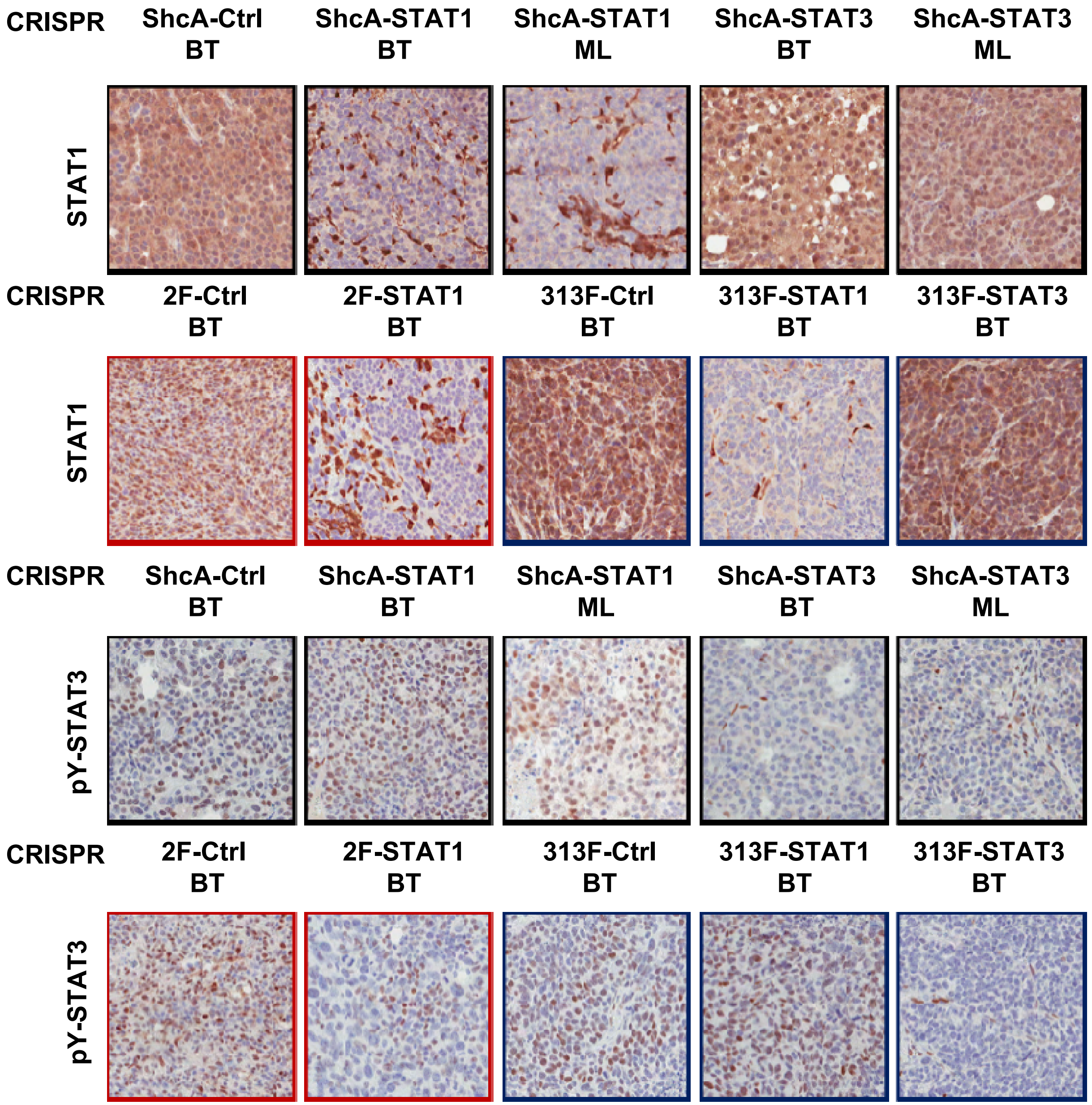
CRISPR
ShcA-Ctrl BT
ShcA-STAT1 BT
ShcA-STAT1 ML
ShcA-STAT3 BT
ShcA-STAT3 ML
STAT1
CRISPR
2F-Ctrl BT
2F-STAT1 BT
313F-Ctrl BT
313F-STAT1 BT
313F-STAT3 BT
STAT1
CRISPR
ShcA-Ctrl BT
ShcA-STAT1 BT
ShcA-STAT1 ML
ShcA-STAT3 BT
ShcA-STAT3 ML
pY-STAT3
CRISPR
2F-Ctrl BT
2F-STAT1 BT
313F-Ctrl BT
313F-STAT1 BT
313F-STAT3 BT
pY-STAT3

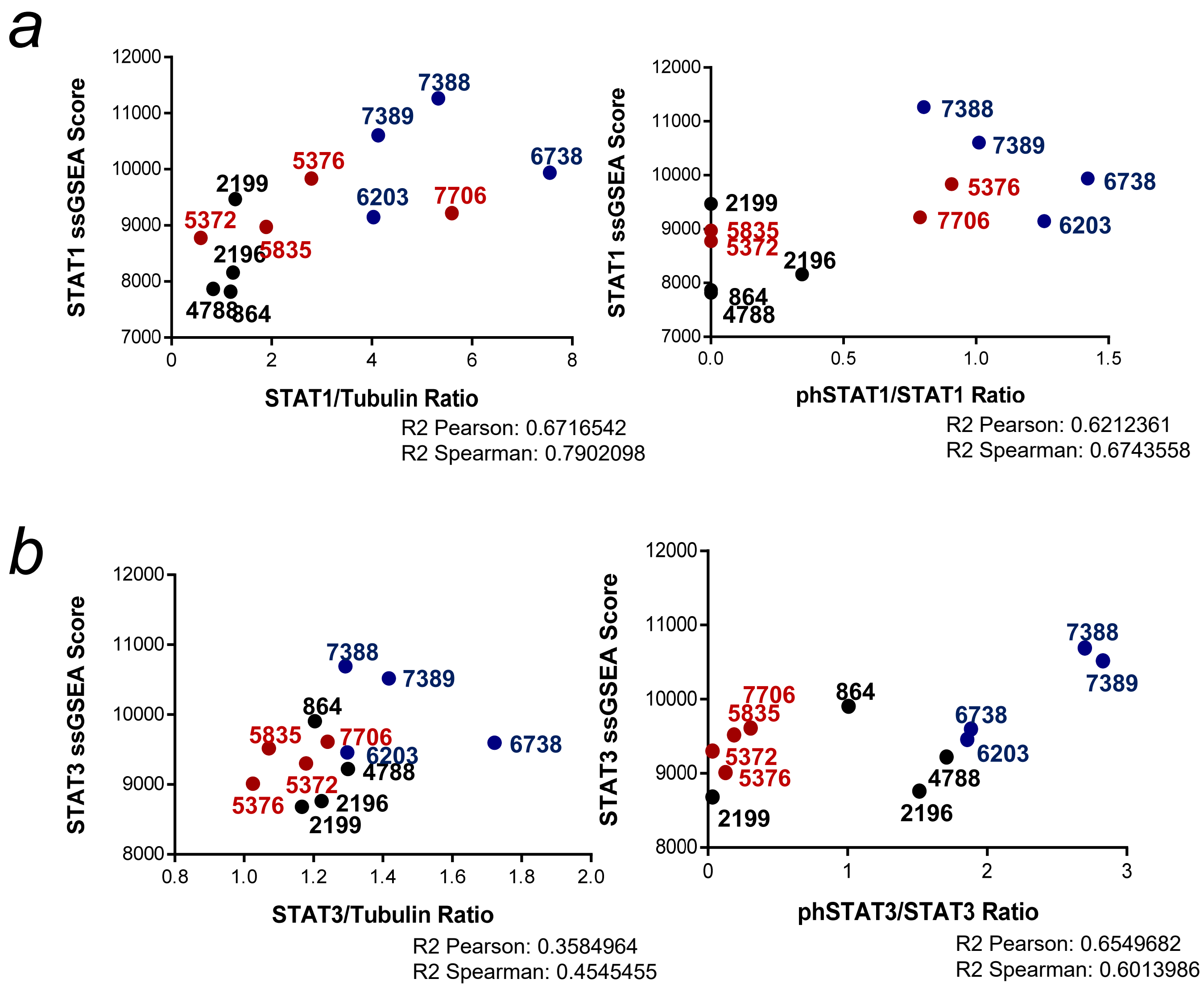

Supplementary Figure 10. STAT1 and STAT3 activation status in mouse mammary tumors correlates with expression levels of their respective transcriptional target genes. Immunoblot analysis was used to measure **(a)** STAT1/Tubulin, pSTAT1/STAT1,**(b)** STAT3/Tubulin and pSTAT3/STAT3 levels from four independent MT/ShcA$^{+/+}$, MT/Shc$^{2F/2F}$ and MT/Shc$^{313F/313F}$ breast cancer cell lines. Quantification of triplicate experiments is shown in Figure 3b and is employed herein to evaluate the relationship between STAT1/3 signaling and expression of target genes. To do so, the STAT1 and STAT3 gene signatures employed to interrogate human breast cancer datasets (Tables S3 and S4) were applied to the RNAseq data generated for each cell line *in vitro* to generate ssGSEA scores. For each cell line, the relationship between STAT1 and STAT3 ssGSEA scores relative to STAT1 and STAT3 expression levels or activation of each pathway (as assessed by tyrosine phosphorylation) was determined.

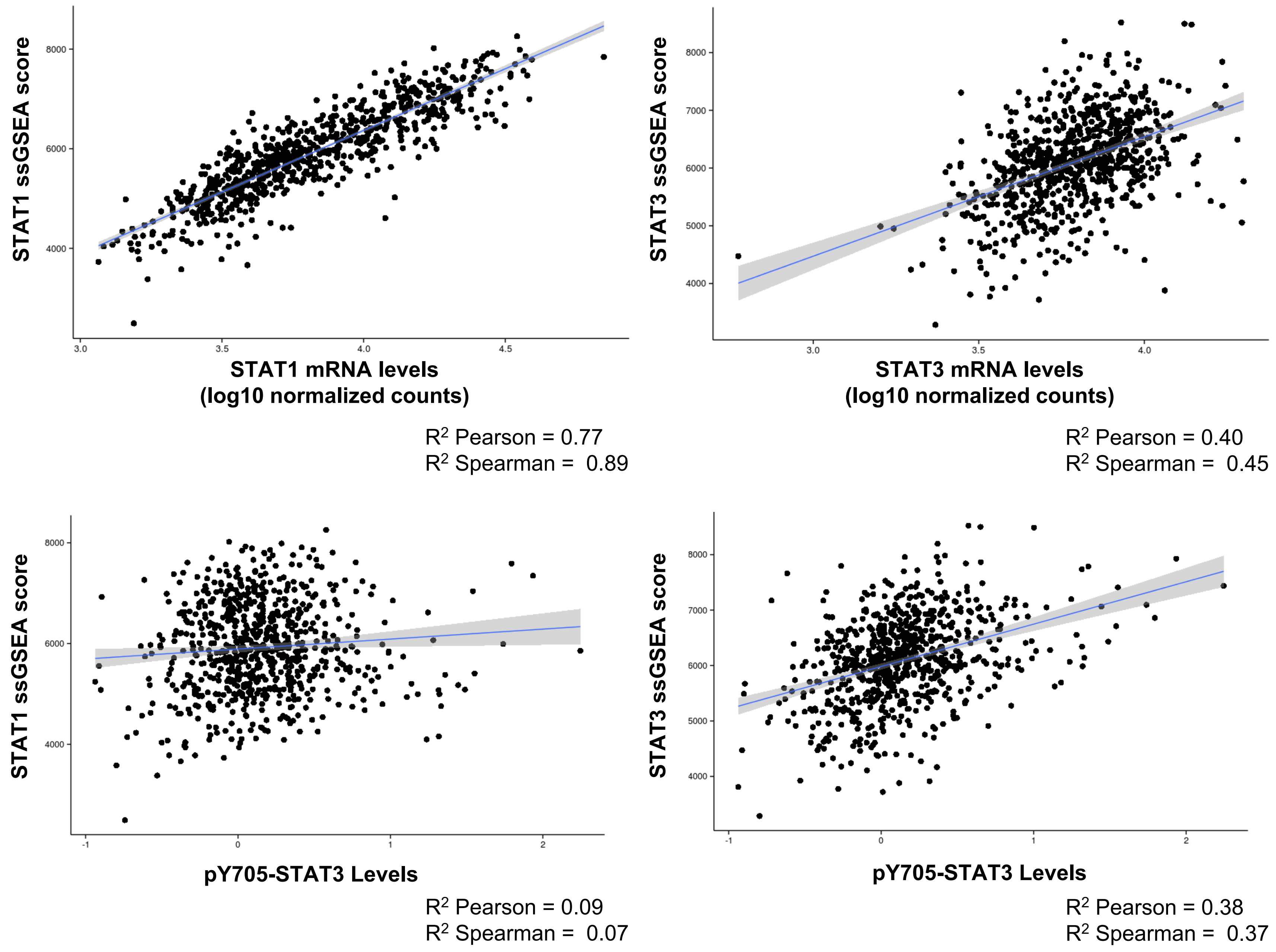

Supplementary Figure 11. STAT1/3 mRNA levels, as well as pY705-STAT3 levels, positively correlate with mRNA levels of their corresponding transcriptional targets. (a) Human breast tumors from the TCGA dataset (N=1215) were evaluated for their relative STAT1 and STAT3 mRNA levels; correlation with the activation of the corresponding gene signature, as measured by ssGSEA score, is shown. (b) For a subset of TCGA breast tumors (N=747), the level of pY705-STAT3 has been determined using reverse phase protein arrays. pY05-STAT3 positively correlates with STAT3 signature, as measured by ssGSEA scores, but not with STAT1 signature, as expected.

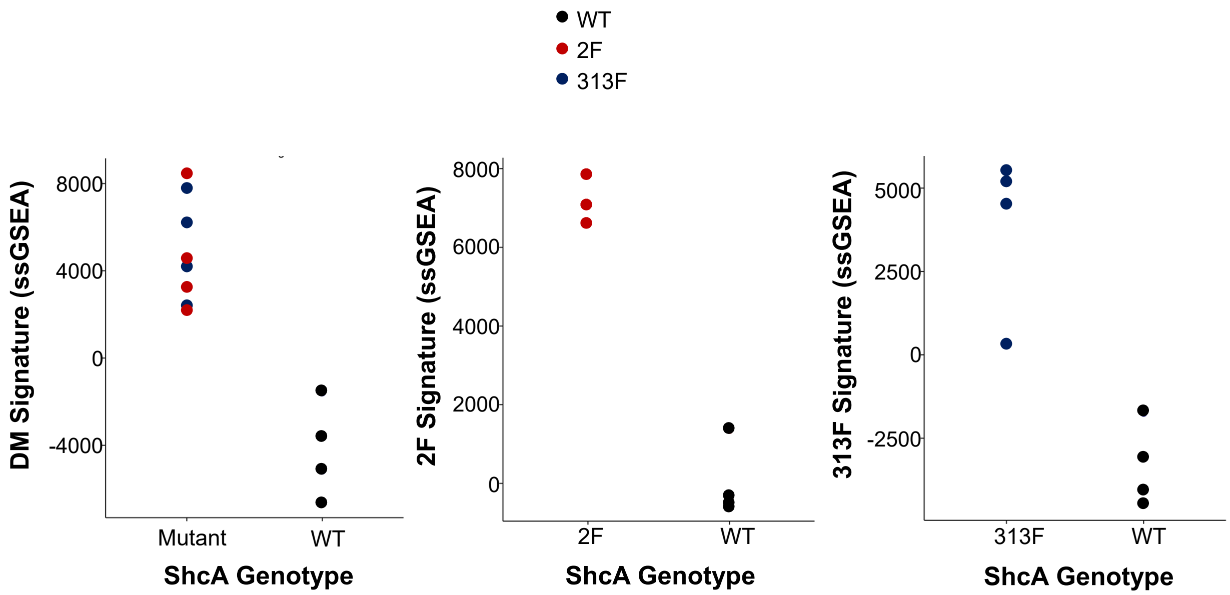

Supplementary Figure 12. The Shc-DM, Shc2F and Shc313F gene signatures employed to stratify human breast cancers accurately predict their respective ShcA genotypes in MT-transformed breast cancer cell lines. Subsets of ShcA-regulated genes (as assessed by RNAseq analysis -Fig. 2) did not have human orthologues and thus were excluded from the Shc-DM, Shc2F and Shc313F gene signatures used to interrogate the TCGA human breast cancer dataset. To verify that the reduced gene signatures (i.e. restricted to genes with human orthologues) maintain their strong association with the different ShcA genotypes, we computed the ssGSEA scores for the four independent MT/ShcA$^{+/+}$, MT/Shc$^{2F/2F}$ and MT/Shc$^{313F/313F}$ breast cancer cell lines that were subjected to RNAseq analysis. Indeed, the double mutant (DM) gene signature is sufficient to discriminate MT/Shc$^{2F/2F}$ and MT/Shc$^{313F/313F}$ cells from MT/ShcA$^{+/+}$ controls, while Shc2F and Shc313F gene signatures can discriminate MT/Shc$^{2F/2F}$ and MT/Shc$^{313F/313F}$ cell lines, respectively, from MT/ShcA$^{+/+}$controls.

Supplementary Figure 13: Uncropped Immunoblots for all data elements shown in the manuscript

Figure 2d: β2M immunoblot

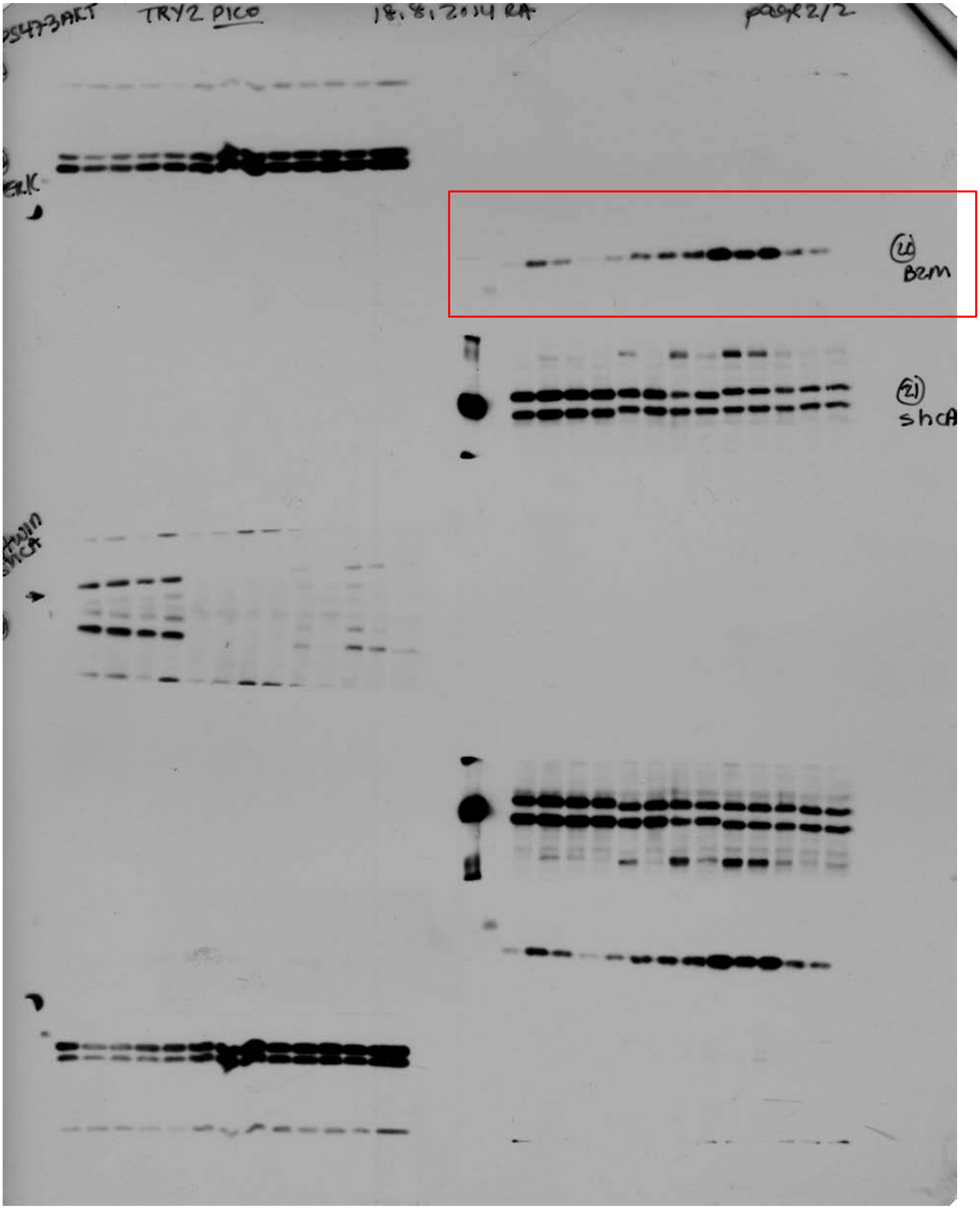

Figure 2d: Tubulin Immunoblot

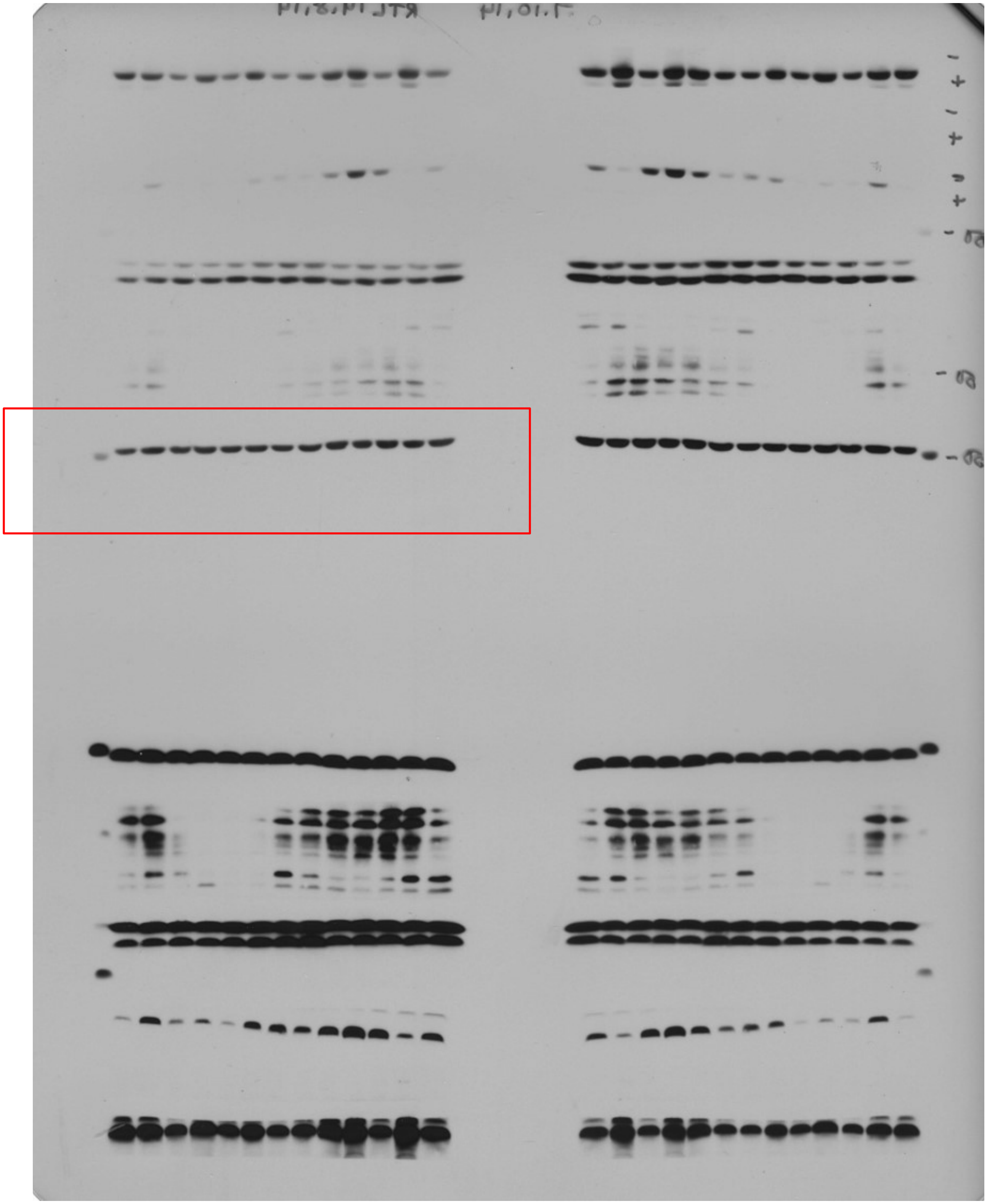

Figure 3a (upper panel): pY701-STAT1 Immunoblot

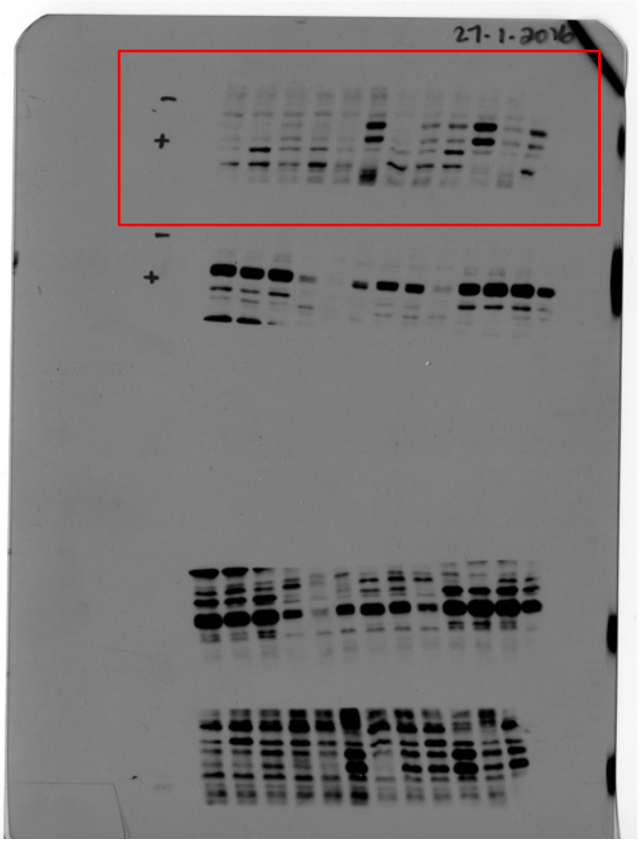

Figure 3a (upper panel): STAT1 Immunblot

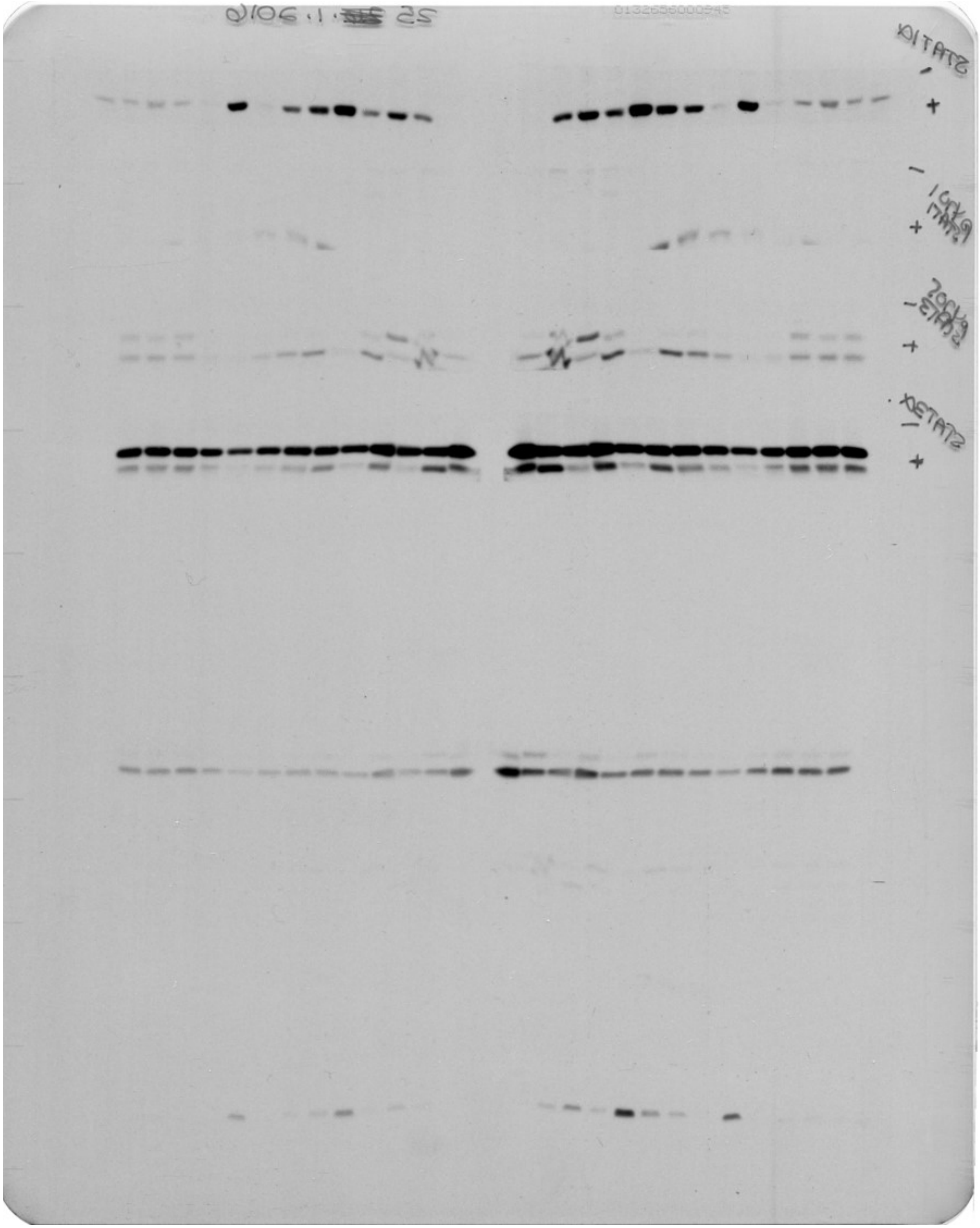

Figure 3a (upper panel): Tubulin Immunoblot

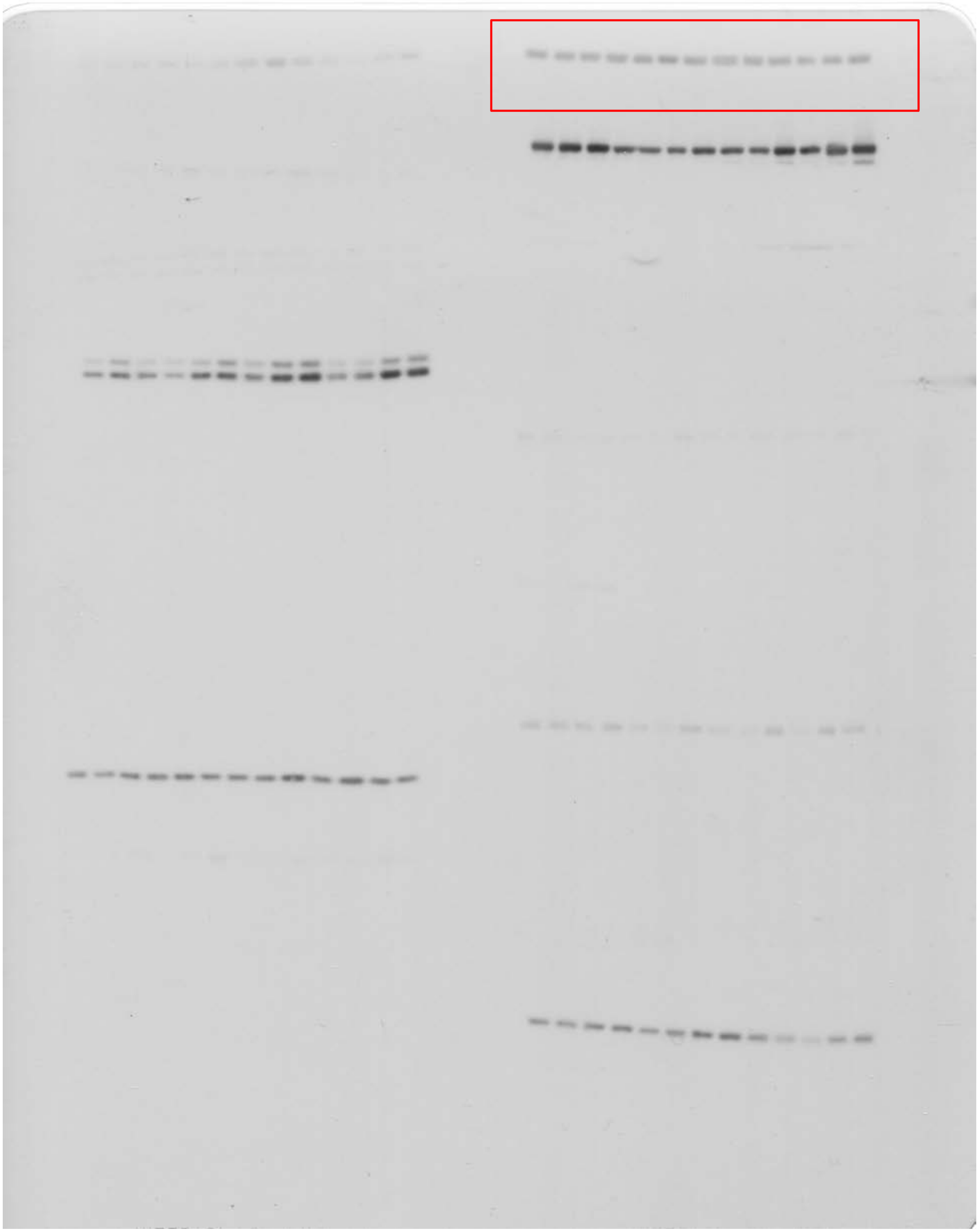

Figure 3a (lower panel): pY705-STAT3 Immunoblot

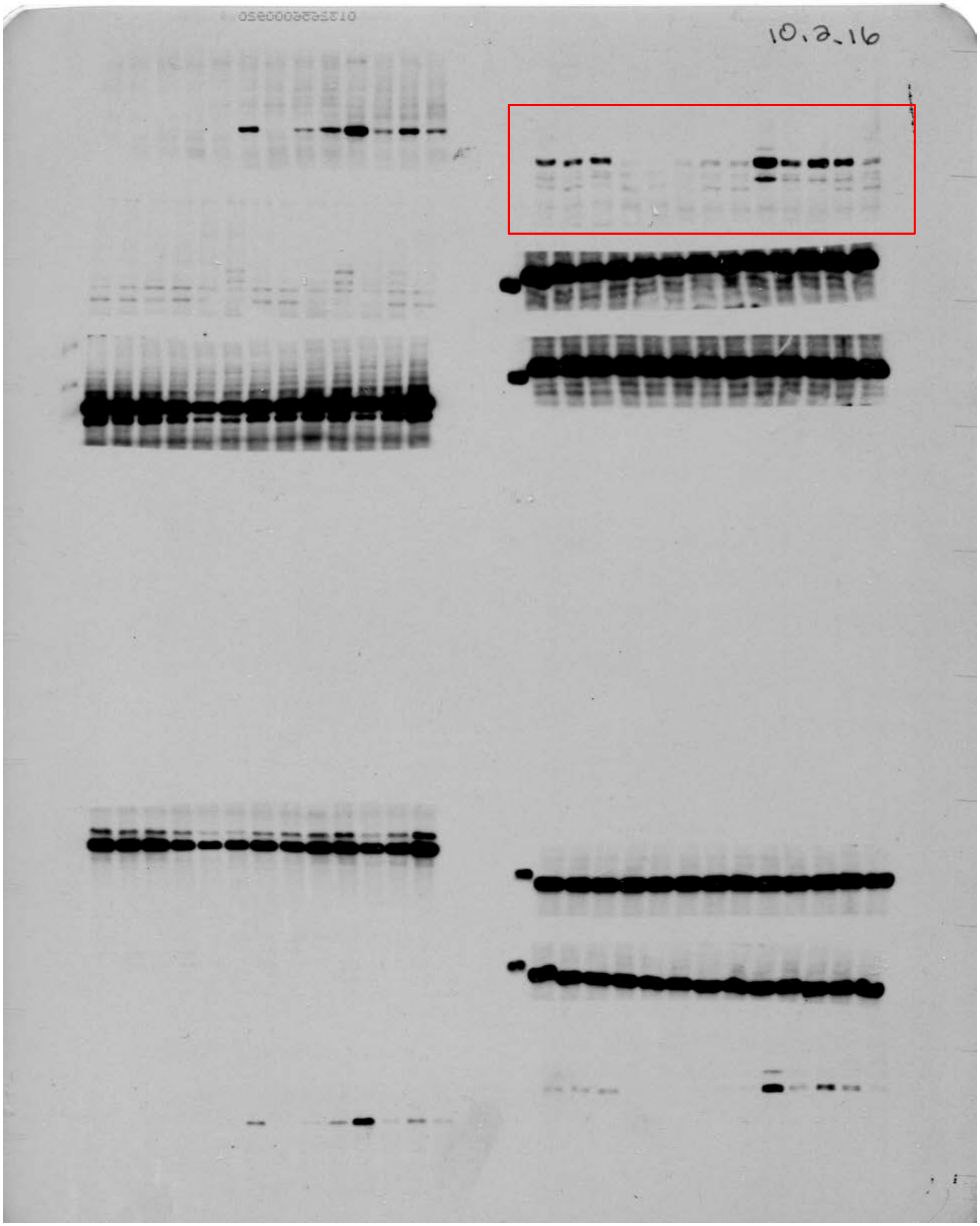

Figure 3a (lower panel): STAT1 Immunoblot

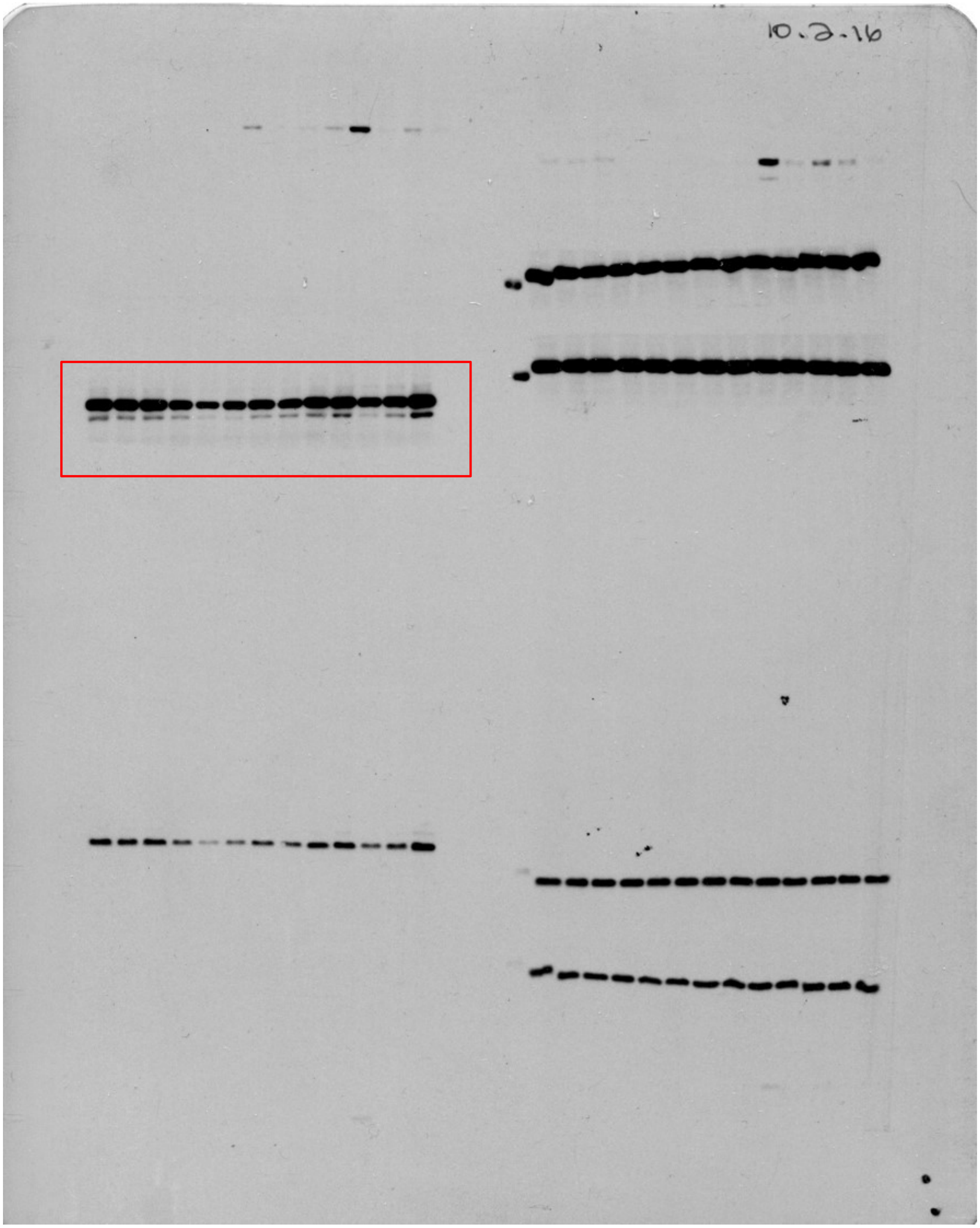

Figure 3a (lower panel): Tubulin Immunoblot

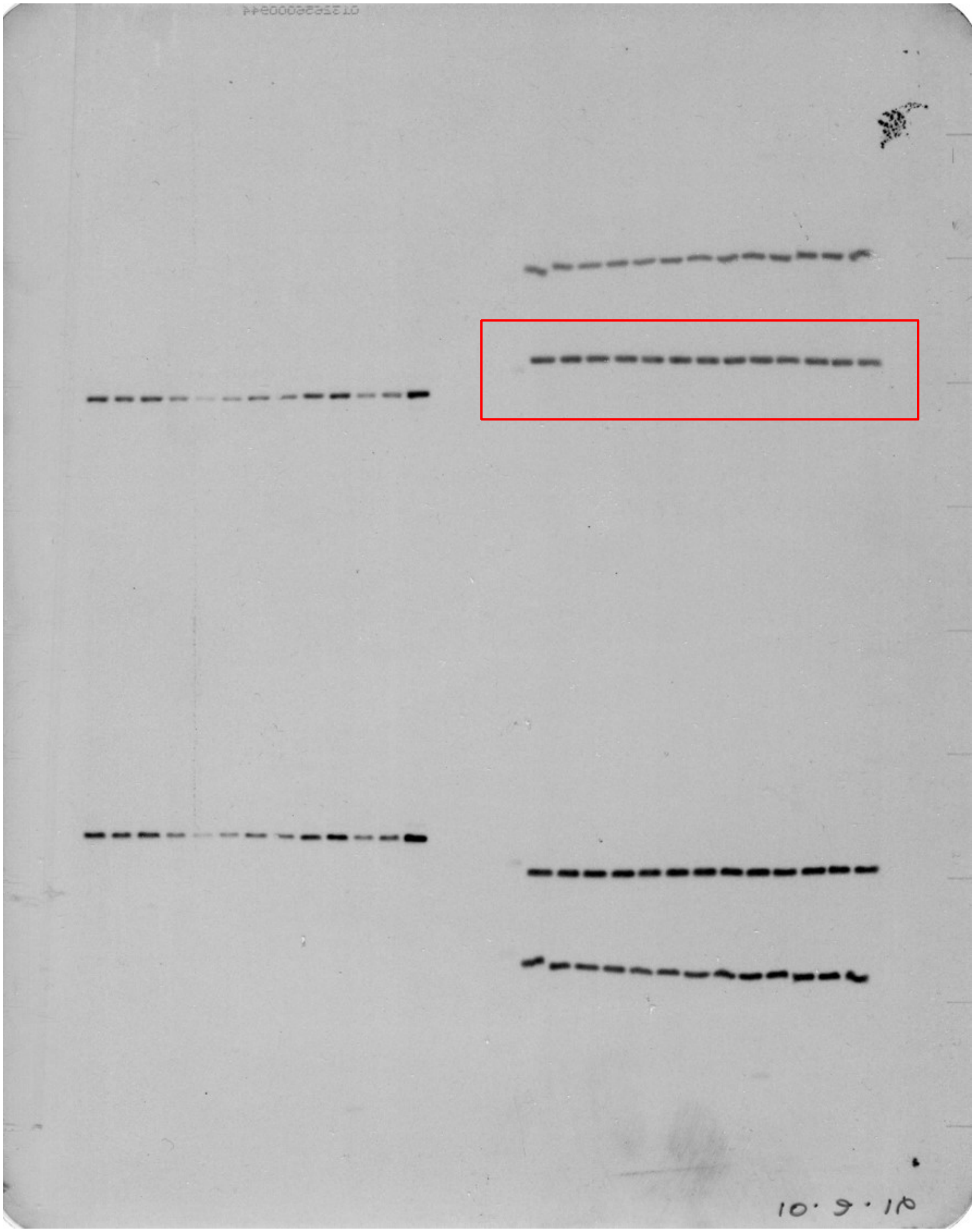

Figure 4a: STAT1 immunoblot

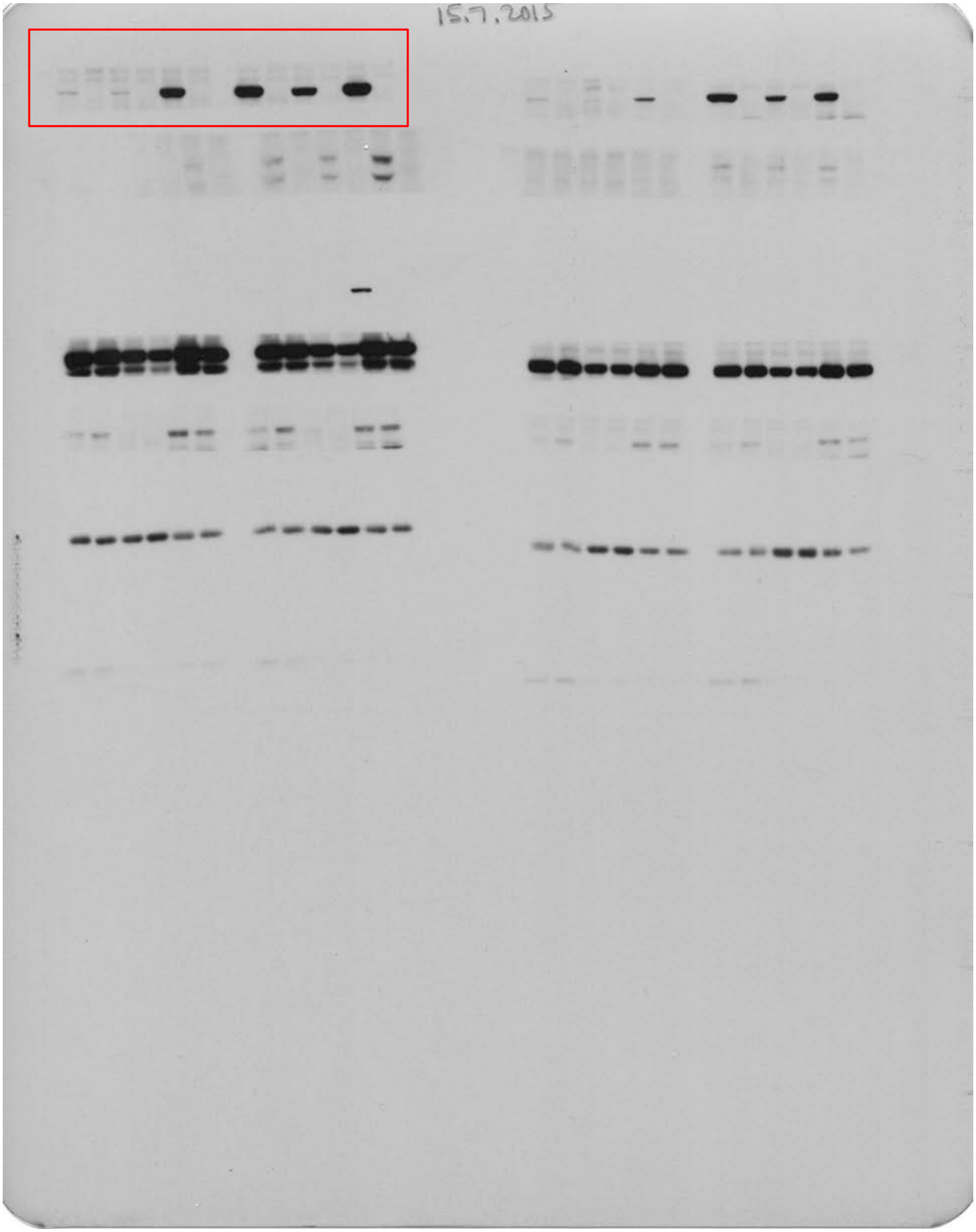

Figure 4a: pY-705 STAT3 and pY-701 STAT1 immunoblots

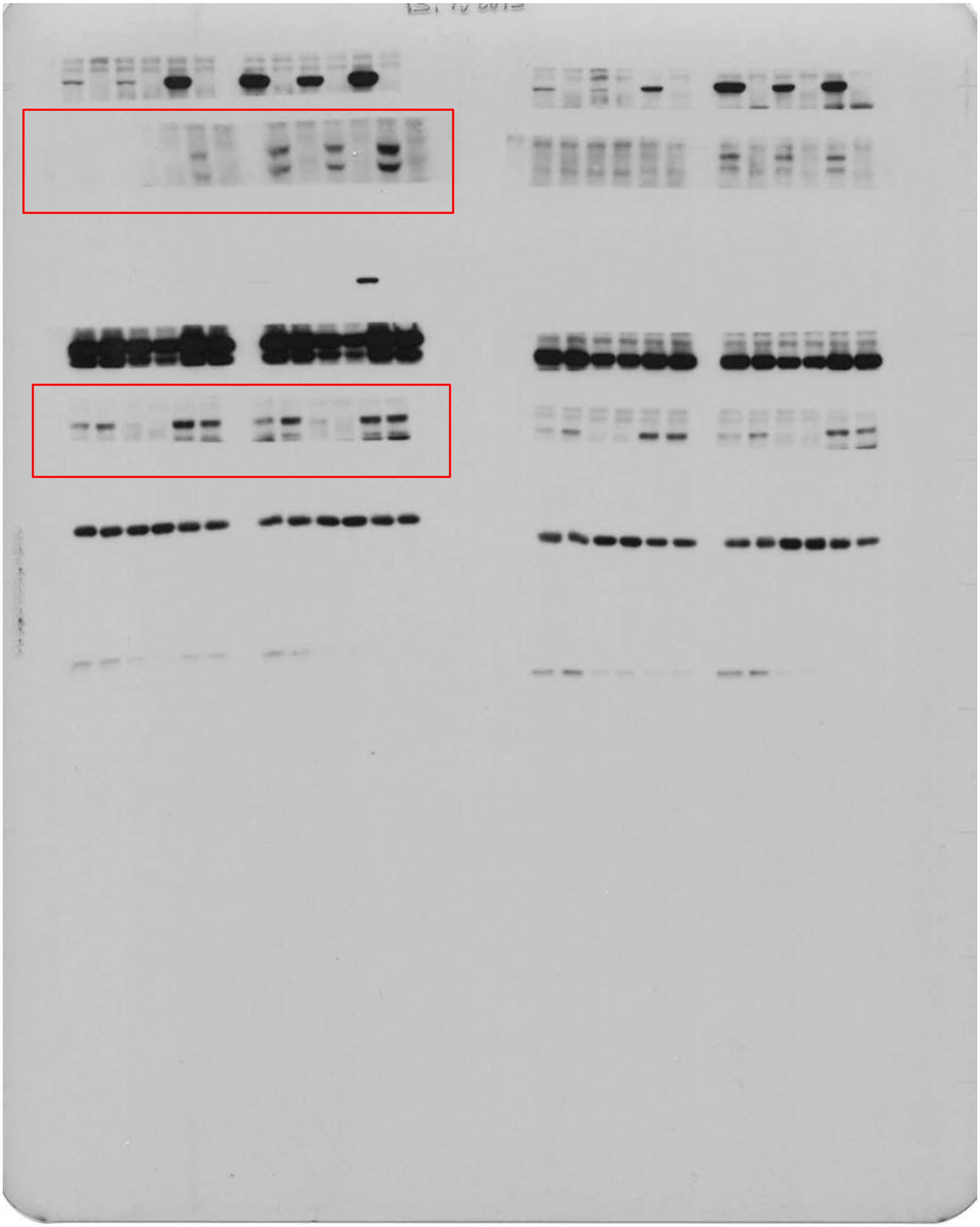

Figure 4a: STAT3 immunoblot

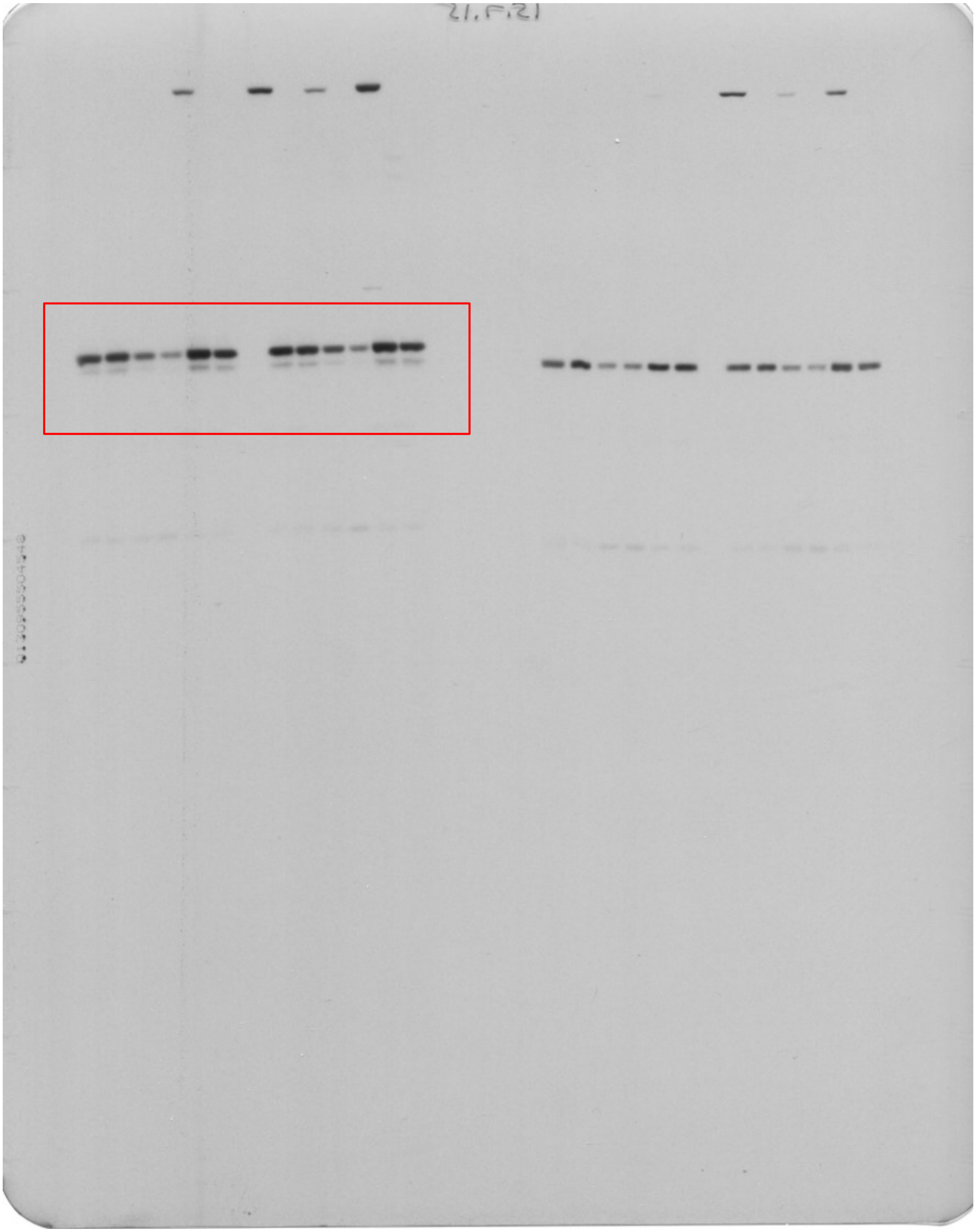

Figure 4a: Tubulin immunoblot

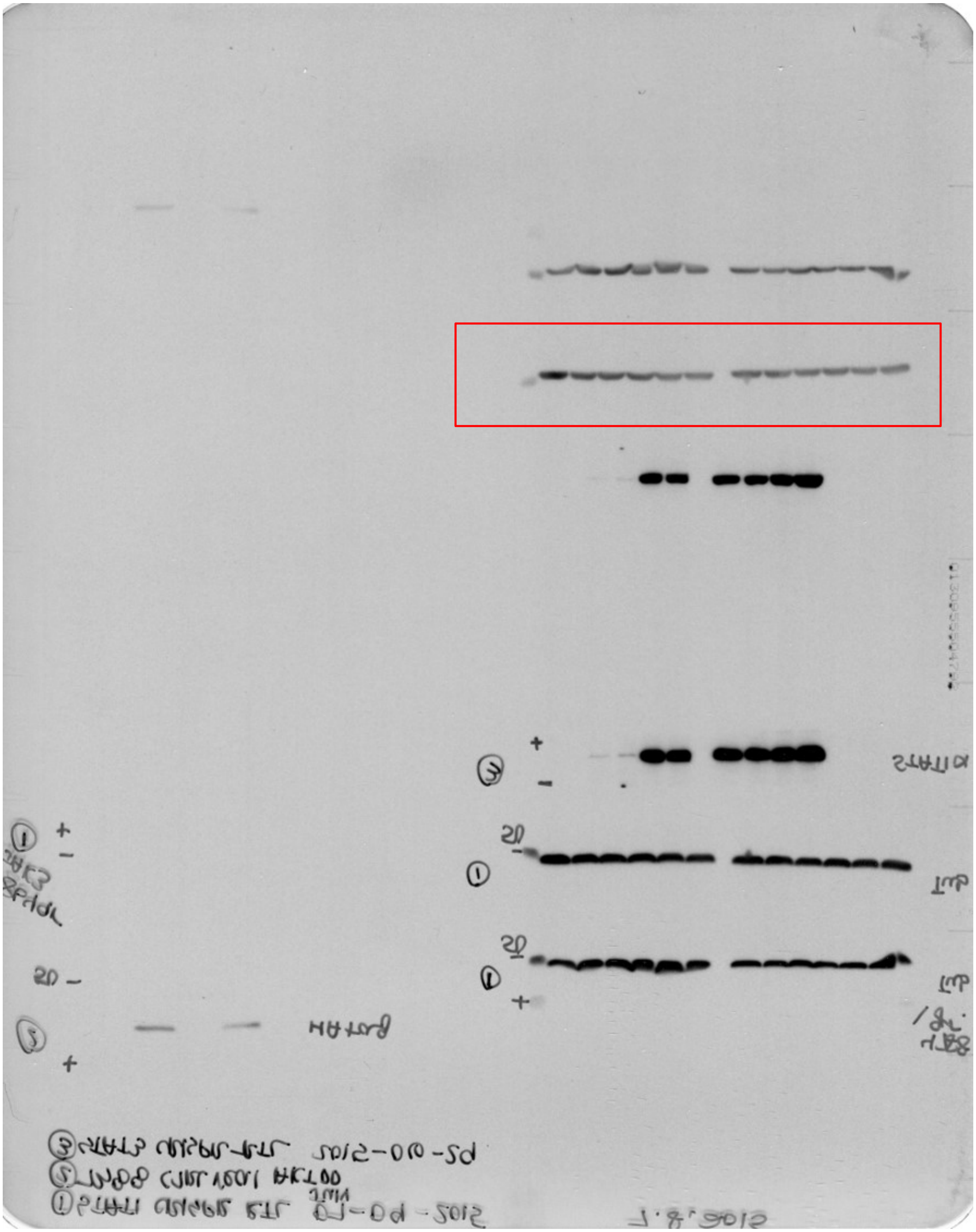

Figure 4b: pY705-STAT3, pY701-STAT1 and Tubulin immunoblots

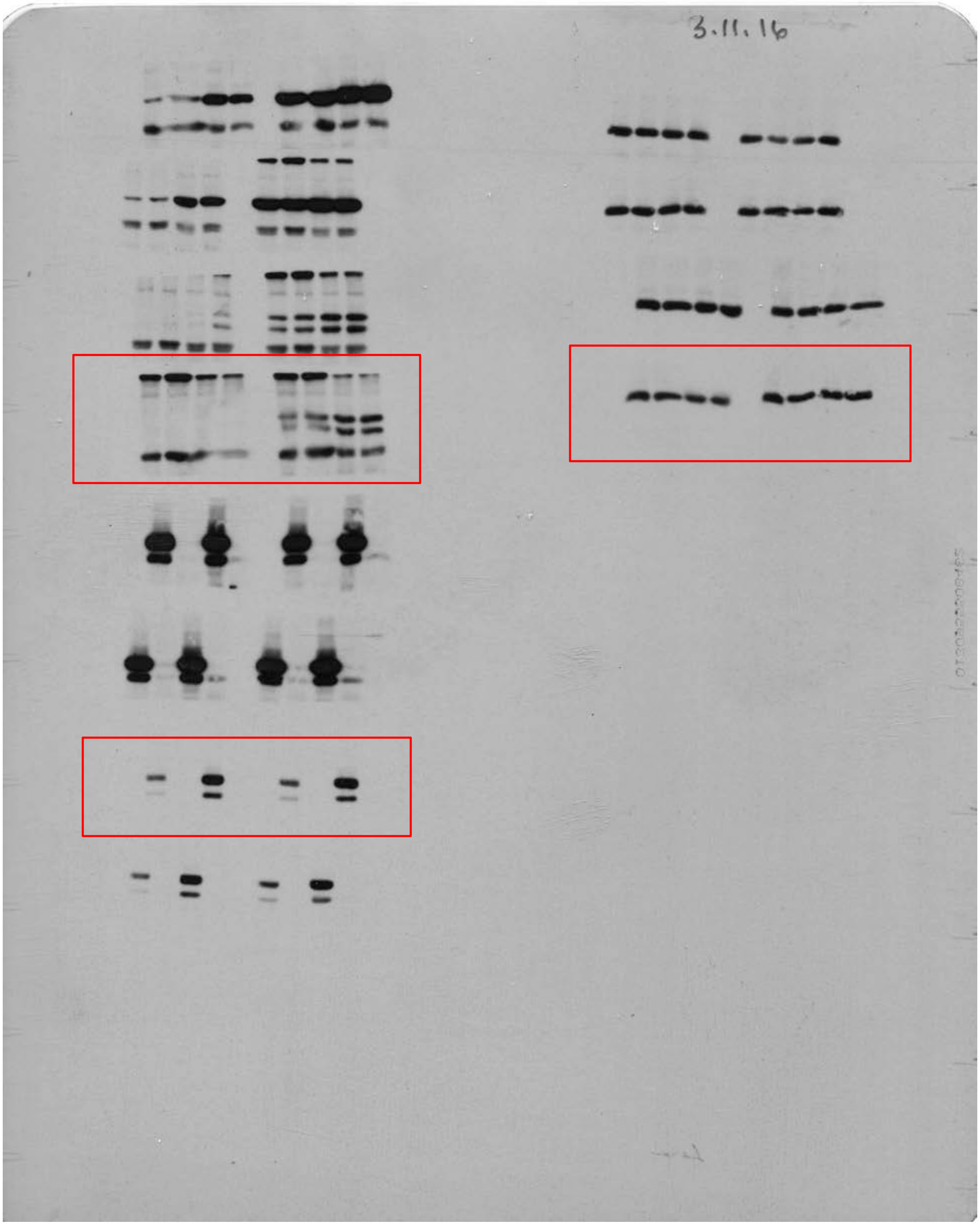

Figure 4b: STAT1 and STAT3 immunoblots

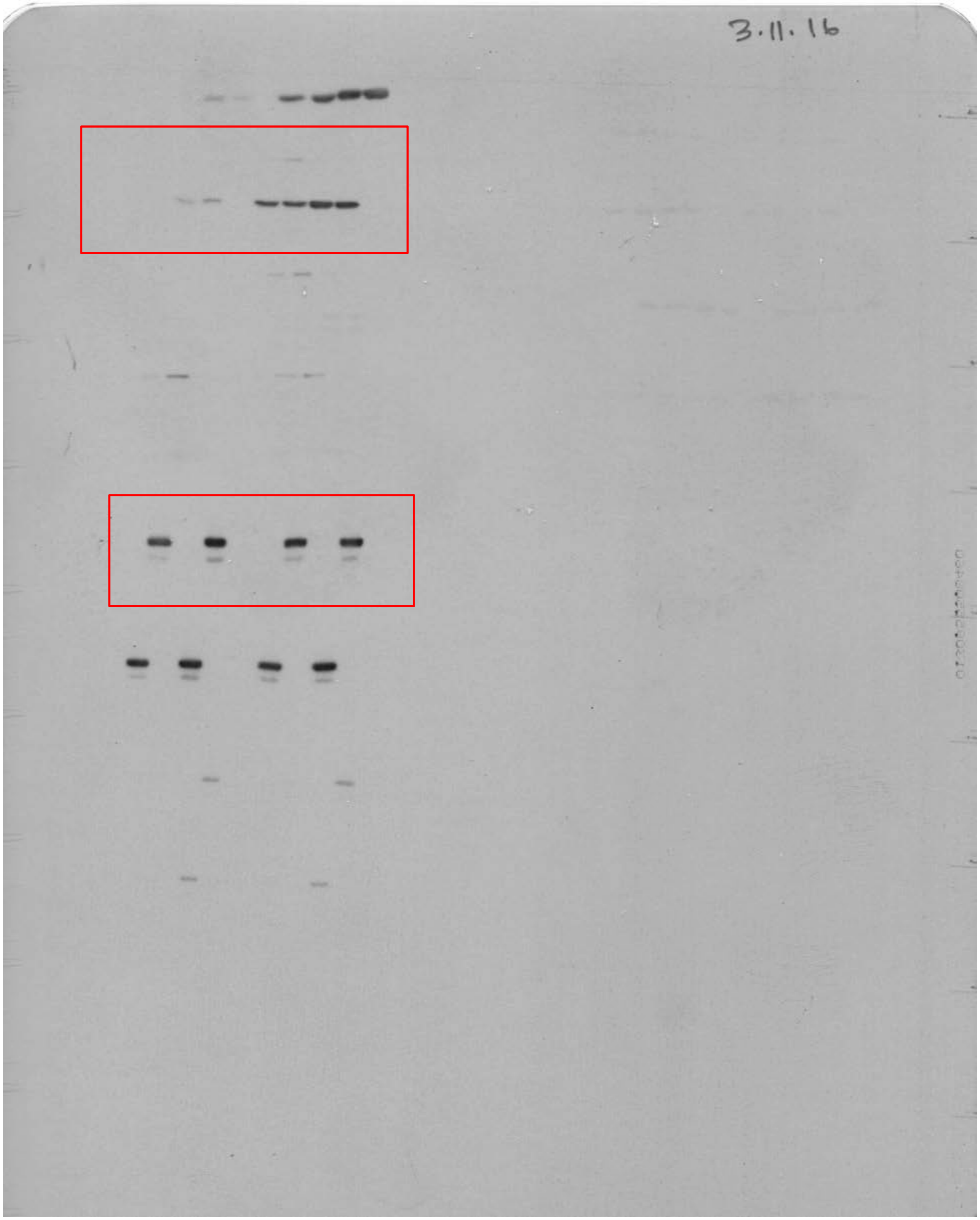

Supplementary Table 1. Expression levels of multiple IFN genes in independent cell lines of the indicated genotypes as determined by the number of reads identified by RNAseq analysis.

	MT/ShcA$^{+/+}$				MT/Shc$^{2F/2F}$				MT/Shc$^{313F/313F}$			
**Gene**	**864**	**2196**	**2199**	**4788**	**5372**	**5376**	**5835**	**7706**	**6203**	**6738**	**7388**	**7389**
Ifna1	0	0	0	0	0	0	0	0	0	0	0	0
Ifna2	0	0	0	0	0	0	0	0	0	0	0	0
Ifna4	0	2	0	0	0	0	0	2	2	2	5	4
Ifna5	0	0	0	0	0	0	0	0	0	0	0	0
Ifna6	0	0	0	0	0	0	0	0	0	0	0	0
Ifna7	0	0	0	0	0	0	0	0	0	0	0	0
Ifna9	0	0	0	0	0	0	0	0	0	0	0	0
Ifna14	0	0	0	0	0	0	0	0	0	0	0	0
Ifnb1	0	0	1	0	0	0	2	0	0	6	7	15
Ifng	0	0	0	0	0	0	0	0	0	0	0	0

Supplementary Table 2. List of STAT1 target genes that define the STAT1 ssGSEA signature.

Gene	Score*	References**
**STAT1**	1	1,3,5,6
**PARP14**	0.83059951	3
**CXCL10**	0.79534805	1
**PARP9**	0.79338464	1,3,4
**TAP1**	0.78190277	1,4
**GBP5**	0.75997402	1
**MX1**	0.71665641	1
**DTX3L**	0.71112409	1,3,4
**GBP1**	0.71097103	1
**NLRC5**	0.68864493	2
**AIM2**	0.68719323	1
**ZBP1**	0.68547864	6
**TAP2**	0.6848176	1
**BATF2**	0.68213951	1,3
**IDO1**	0.67601428	1
**CXCL9**	0.67269701	1
**PSMB9**	0.65108485	1
**IRF9**	0.64794506	1,2,4,5
**PLSCR1**	0.64664812	1,3,5
**APOL6**	0.63641934	1,3
**CCRN4L**	0.63529631	1,2
**APOL1**	0.62604301	1
**IFI27**	0.6189761	1,4
**TRIM21**	0.58228556	1
**IRF1**	0.57598741	1,2,3,5
**NMI**	0.56423296	1,4
**PSMB8**	0.55738005	1,4
**PDCD1LG2**	0.54932666	1,2
**RTP4**	0.53875957	4
**CIITA**	0.4689531	1
**BRCA2**	0.45651856	1,3
**APOL3**	0.44997339	1
**CXCL13**	0.44906025	1,6
**WIPF1**	0.44532865	1,2
**IFI35**	0.43718503	1,3,4
**BST2**	0.43643893	1,3
**TMEM140**	0.43144908	1,4
**LGALS3BP**	0.42831041	1,4
**IDO2**	0.42663042	1
**IFI16**	0.38574132	1,3,5
**IL15RA**	0.36595285	1,6
**MTHFD2**	0.34958656	1,2,3
**TOP1**	0.34441541	1,2
**KIF2A**	0.34239429	1,2,6
**PML**	0.33822912	1,2
**MFHAS1**	0.33226027	2
**IRF7**	0.325158	1,5

**BCAT1**	0.32212846	1
**ERAP1**	0.32094206	4
**EZH2**	0.3108083	1,2
**CAND1**	0.29682544	2,4
**GBP2**	0.28108758	1,4
**SETX**	0.27760148	2,6
**CASP7**	0.26854694	1,2
**PHEX**	0.26788217	1
**RAP1A**	0.26093333	1,2,3
**IL10RB**	0.24953463	1
**NUP210**	0.23707301	2,3
**DNMT3B**	0.2259721	1
**GLRX**	0.22103016	2
**COPG**	0.21878909	1,3
**CCDC60**	0.21652676	1
**UHMK1**	0.21045863	1,2
**CLIC2**	0.20880268	1,3
**HM13**	0.18924991	1,2,3
**CYP1B1**	0.18806773	1,2
**UEVLD**	0.16493473	1,2
**TRIM25**	0.16334499	1
**NCOA7**	0.1570198	1,2
**FYN**	0.15238024	1
**CSF1**	0.14813757	1,2
**APAF1**	0.14155321	1,2
**USP3**	0.13756893	2
**RDX**	0.13545045	1,2,6
**ZNF473**	0.13393581	1,2,3
**IRF2**	0.13307439	1,2,5
**IPO8**	0.12870036	1,2,3
**GFM1**	0.12783804	1,2,3
**KLF3**	0.12780589	1,2,5
**ATMIN**	0.12195655	2,6
**RDH10**	0.11789259	1,2
**IMPAD1**	0.11617159	1,2
**NOX4**	0.11604627	1,2
**BAZ2A**	0.11316689	1,2,3
**UBP1**	0.11201484	1,2,5
**CCDC6**	0.11092008	1,2
**UBR1**	0.11078076	1,2,3
**MORC3**	0.10306528	1,2
**HRH1**	0.1013663	1,2

*The frequency with which relative expression levels of each gene are comparable with STAT1 mRNA levels in individual breast tumors from the TCGA RNAseq dataset (n=1215).

**Refer to the Supplementary References at the end of the Supplementary Information file

Supplementary Table 3. List of STAT3 target genes that define the STAT3 ssGSEA signature.

Gene	Score*	References**
**STAT3**	1	4,7,9,10
**IL6ST**	0.43835358	4,8
**NFKB1**	0.41621436	9
**FOSL2**	0.41340799	7,9
**SLC4A7**	0.35683991	7
**UGCG**	0.33030354	7,8
**MCL1**	0.30895228	6,8,11
**PFKFB3**	0.30336005	7
**JAK2**	0.27602273	7,11
**FEM1C**	0.27566194	7
**SAMD4A**	0.27486799	7
**AKAP2**	0.26924854	7
**AKAP12**	0.26716036	7,8
**STOM**	0.25314173	7
**ICAM1**	0.25181764	7
**ABCA1**	0.25120452	7
**FLNB**	0.25015441	7
**MBNL2**	0.2470801	4,7
**SERPINA3**	0.2463125	4,7,8,10
**TGFB2**	0.24187624	13
**SOD2**	0.22952525	7
**THBS1**	0.2255324	7,8
**BCL6**	0.21904633	7,9,11
**C3**	0.20114834	7
**FGL2**	0.19811681	13
**TEK**	0.19164484	13
**THBD**	0.19113941	7,12
**MUC1**	0.18890754	8
**NPC1**	0.18613671	7,8
**LAMA3**	0.17806808	7
**GFPT2**	0.17546918	7
**LDLR**	0.17146452	7,8
**PPAP2B**	0.16594436	7
**PELI2**	0.16552106	7
**CCND2**	0.16316837	12
**FGB**	0.1544856	7
**LBP**	0.15409878	7,10
**AGT**	0.14964365	7,8
**A2M**	0.14909279	8,10
**TRIB1**	0.14849039	15
**SLC2A14**	0.14756321	7
**MMP2**	0.13652352	6,8
**SOCS3**	0.13617275	6,7,8,10,11,14
**ATF3**	0.13325996	7,8,9
**SLC2A3**	0.12961652	7,8

**FOS**	0.12886529	8,9,10
**FGG**	0.12710997	7,10
**ZFP36**	0.12528609	7
**KLF11**	0.1048312	10
**PLOD2**	0.10282667	7
**IL6**	0.10095934	4,14

*The frequency with which relative expression levels of each gene are comparable with STAT1 mRNA levels in individual breast tumors from the TCGA RNAseq dataset (n=1215).

**Refer to the Supplementary References at the end of the Supplementary Information file

Supplementary Table 4. List of antibodies employed for immunoblotting in this study.

Epitope	Cat #	Company	Dilution
**β2 microglobulin**	ab75853	Abcam	1:15,000
**STAT1 pY701**	9171	Cell Signaling	1:1,000
**STAT1a (C-111)**	Sc-417	Santa Cruz	1:2,000
**STAT3 (124H6)**	9139	Cell Signaling	1:2,000
**STAT3 pY705 (D3A7)**	9145	Cell Signaling	1:1,000
**Tubulin**	T5168	Sigma Aldrich	1:15,000

Supplementary Table 5. List of antibodies employed for immunohistochemistry in this study.

Epitope	Cat #	Company	Dilution	Tissue	Antigen retrieval[a]
**STAT1a**	SC-417	Santa Cruz	1:750	Paraffin-embedded	Sodium Citrate Buffer
**STAT3 pY705**	9145	Cell Signaling	1:200	Paraffin-embedded	TE buffer
**CD3**	ab16669	abcam	1:200	Paraffin-embedded	Sodium Citrate Buffer
**Granzyme B**	ab4059	Cedarlane	1:200	Paraffin-embedded	Sodium Citrate Buffer

[a] Composition of Sodium Citrate Buffer (1X): 10mM Sodium Citrate (2.94g Tri-sodium citrate (dihydrate) in 1000ml distilled water. Adjust pH to 6.0 with 1N HCl) with 0.05% Tween 20; Composition of TE Buffer (1X): 1.21g Tris and 0.37g EDTA in 1000ml distilled water (pH 9.0) with 0.05% Tween 20.

Supplementary Table 6. List of Antibodies and Reagents Used for Flow Cytometry

Epitope	Fluorophore	Cat#	Company	[Ab] Tumor	[Ab] Spleen
**Primary Ab**					
**B220**	Alexa-488	557669	BD Pharmingen	0.2 µg	0.1 µg
**CD8a**	APC	47-0081-82	Ebioscience	0.1 µg	0.05 µg
**CD69**	PE	553237	BD Pharmingen	0.4 µg	0.2 µg
**Gr1 (Ly6G)**	Alexa-488	53-5931-82	Ebioscience	6.25 ng	2.125 ng
**CD11b**	APC	17-0112-81	Ebioscience	2.5 ng	1.25 ng
**CD45**	BV785	103149	Biolegend	0.8 µg	0.4 µg
**Reagents**					
**Mouse Fc Block (CD16/CD32)**	N/A	553142	BD Pharmingen	2 µg	1 µg
**Live/Dead Fixable**	Aqua	L34960	Invitrogen	0.6 µl into 49.4 µl PBS	0.3 µl into 24.7 µl PBS

Supplementary Table 7. **List of RT-qPCR primers used for murine genes.**

Genes	SYBR Green[a]		Taqman probe[b]
	Forward sequence (5'-3')	Reverse sequence (5'-3')	
**ACTB**	GGCTGTATTCCCCTCCATCG	CCAGTTGGTAACAATGCCATGT	
**B2M**	TGGTCTTTCTGGTGCTTGTCT	ATTTTTTTCCCGTTCTTCAGC	
**CD274**	GCTCCAAAGGACTTGTACGTG	TGATCTGAAGGGCAGCATTTC	
**CXCL9**	GGAGTTCGAGGAACCCTAGTG	GGGATTTGTAGTGGATCGTGC	
**DDX60**	TTCCACTGCCCAAAATAGGAAAA	GCCAGCAACATGAGTCTTAGGAT	
**ERAP1**	TAATGGAGACTCATTCCCTTGGA	AAAGTCAGAGTGCTGAGGTTTG	
**GAPDH**	AACGACCCCTTCATTGAC	TCCACGACATACTCAGCAC	
**IFNG**	TGTGGCCTAATTACTCATGCTC	ATGGAAAGGCAGAAGCAAAGT	
**IRF9**	GCCGAGTGGTGGGTAAGAC	GCAAAGGCGCTGAACAAAGAG	
**MUC1**	TCGTCTATTTCCTTGCCCTG	ATTACCTGCCGAAACCTCCT	
**PRF1**	GGTTTTTGTACCAGGCGAAA	GATGTGAACCCTAGGCCAGA	
**PSMB8**	ATGGCGTTACTGGATCTGTGC	CGCGGAGAAACTGTAGTGTCC	
**TBP**	ACCTTATGCTCAGGGCTTGG	GCCATAAGGCATCATTGGAC	
**TAP1**			Mm00443188_m1
**TAP2**			Mm01277033_m1

[a] goTaq SYBR Green Mix from ThermoFisher (Cat# PRA6002). [b] Purchased from ThermoFisher Scientific and used with Taqman MasterMix 2x (Cat#4352042; Life Technologies)

Supplementary References

1. Satoh, J., and Tabunoki, H. (2013). A Comprehensive Profile of ChIP-Seq-Based STAT1 Target Genes Suggests the Complexity of STAT1-Mediated Gene Regulatory Mechanisms. Gene regulation and systems biology *7*, 41-56.
2. Wang, G., Wang, Y., Teng, M., Zhang, D., Li, L., and Liu, Y. (2010). Signal transducers and activators of transcription-1 (STAT1) regulates microRNA transcription in interferon gamma-stimulated HeLa cells. PLoS One *5*, e11794.
3. Bhinge, A. A., Kim, J., Euskirchen, G. M., Snyder, M., and Iyer, V. R. (2007). Mapping the chromosomal targets of STAT1 by Sequence Tag Analysis of Genomic Enrichment (STAGE). Genome Res *17*, 910-916.
4. TFsearcher (http://diyhpl.us/~bryan/irc/protocol-online/protocol-cache/TFSEARCH.html)
5. Chawla, K., Tripathi, S., Thommesen, L., Laegreid, A., and Kuiper, M. (2013). TFcheckpoint: a curated compendium of specific DNA-binding RNA polymerase II transcription factors. Bioinformatics *29*, 2519-2520.
6. QIAGEN 2015 (http://www.sabiosciences.com/chipqpcrsearch.php?app=TFBS)
7. Dauer, D. J., Ferraro, B., Song, L., Yu, B., Mora, L., Buettner, R., Enkemann, S., Jove, R., and Haura, E. B. (2005). Stat3 regulates genes common to both wound healing and cancer. Oncogene *24*, 3397-3408.
8. Oh, Y. M., Kim, J. K., Choi, Y., Choi, S., and Yoo, J. Y. (2009). Prediction and experimental validation of novel STAT3 target genes in human cancer cells. PLoS One *4*, e6911.
9. Chawla, K., Tripathi, S., Thommesen, L., Laegreid, A., and Kuiper, M. (2013). TFcheckpoint: a curated compendium of specific DNA-binding RNA polymerase II transcription factors. Bioinformatics *29*, 2519-2520.
10. Ehret, G. B., Reichenbach, P., Schindler, U., Horvath, C. M., Fritz, S., Nabholz, M., and Bucher, P. (2001). DNA binding specificity of different STAT proteins. Comparison of in vitro specificity with natural target sites. J Biol Chem *276*, 6675-6688.
11. Alvarez, J. V., Febbo, P. G., Ramaswamy, S., Loda, M., Richardson, A., and Frank, D. A. (2005). Identification of a Genetic Signature of Activated Signal Transducer and Activator of Transcription 3 in Human Tumors. Cancer Research *65*, 5054-5062.
12. Paz, K., Socci, N. D., van Nimwegen, E., Viale, A., and Darnell, J. E. (2004). Transformation fingerprint: induced STAT3-C, v-Src and Ha-Ras cause small initial changes but similar established profiles in mRNA. Oncogene *23*, 8455-8463.
13. Snyder, M., Huang, X. Y., and Zhang, J. J. (2008). Identification of novel direct Stat3 target genes for control of growth and differentiation. J Biol Chem *283*, 3791-3798.
14. Przanowski, P., Dabrowski, M., Ellert-Miklaszewska, A., Kloss, M., Mieczkowski, J., Kaza, B., Ronowicz, A., Hu, F., Piotrowski, A., Kettenmann, H., *et al.* (2014). The signal transducers Stat1 and Stat3 and their novel target Jmjd3 drive the expression of inflammatory genes in microglia. Journal of molecular medicine *92*, 239-254.
15. Marie, C.S., Verkerke, H.P., Paul, S.N., Mackey, A.J. and Petri Jr. W.A. (2012). Leptin protects host cells from Entamoeba histolytica cytotoxicity by a STAT3-dependent mechanism. Infection and Immunity *80*, 1934-1943.
